# Is exposure to online misogyny associated with mental health outcomes in girls and young women? A systematic review of longitudinal studies

**DOI:** 10.1177/17455057261485065

**Published:** 2026-09-23

**Authors:** Ciara Thomas, Laura E Tinner, Jacks Bennett, Lucy G Anderson, Lizzy Winstone

**Affiliations:** 1 Population Health Sciences, Bristol Medical School, University of Bristol, Bristol, UK

**Keywords:** adolescence, mental health, internet, online social networking, sexual harassment, sexism, online misogyny

## Abstract

**Introduction:**

The mental health gender gap is well established, but there is limited understanding of why girls and young women consistently report higher rates of common mental health conditions. The emergence of online misogyny has garnered attention in recent years, highlighted in cultural debate surrounding extremist influencers and the ‘manosphere’. Understanding the mental health impacts of online misogyny on girls, young women and gender-diverse people is essential given the growing prevalence of gender-based harm in digital spaces.

**Objectives:**

To establish whether exposure to online misogyny is longitudinally associated with mental health outcomes in girls, young women and gender-diverse people.

**Design:**

Systematic review with narrative synthesis.

**Data sources and methods:**

We searched PsycINFO, Web of Science, PubMed and Europe PMC (4^th^ September 2025). Eligible studies were longitudinal and assessed mental health outcomes in participants aged 11-25 following exposure to online misogyny. Findings were synthesised narratively. Risk of bias was assessed using the Newcastle-Ottawa Scale.

**Results:**

Six studies were included (total N=7,379), reflecting a small and methodologically diverse body of longitudinal research that provides preliminary support of prospective associations between online pornography exposure or cyber-sexual harassment and mental health outcomes among adolescent and young adult women. We did not identify any studies focused on gender-diverse people.

**Conclusion:**

Given the scale of exposure to online misogyny globally, there is an urgent need for longitudinal, mechanism-focused research that treats online misogyny as a social determinant of mental health. Future studies should explore the impact of non-targeted, pervasive or incidental exposure to misogynistic content, such as the ‘manosphere’, given its prevalence among girls and young women. Such work is essential to inform gender-sensitive clinical guidance, targeted prevention efforts, and platform regulation aimed at reducing psychological harm.

**Registration:**

This review was preregistered on the PROSPERO database (CRD420251125049).

## Introduction

The mental health gender gap is well established, with evidence that common mental health conditions such as anxiety, depression, self-harm and eating disorders, are consistently reported in girls and young women at significantly higher rates than in boys.^1–5^ Gender differences emerge during early to middle adolescence and peak in late adolescence; by the age of 18 girls are more than twice as likely to experience internalising symptoms (for example anxiety and depression) compared to boys.^6,7^ There is still limited understanding of the mental health gender gap; potential psychological, biological, social and economic factors have been proposed to contribute, such as gender roles, expectations and inequality, familial factors, specific vulnerability factors and personal traits, academic pressures, body image, sexual violence, peer relationships and digital environments.^8–12^

An increasingly prominent feature of digital environments is the proliferation of online misogyny, reflected in growing public concern about extremist influencers and the ‘manosphere’.^13^ Online misogyny refers to digital content and behaviours that express or encourage hatred, abuse, or sexually harmful actions directed at women and girls.^14,15^ These harms can include exposure to misogynistic or hypermasculine content, online sexual harassment, image-based abuse, coercive sexual communication, sexist harassment, and some forms of mainstream online pornography that reinforce gendered power inequalities or sexual aggression towards women.^16,17^ Recent work has argued that such experiences should be conceptualised not merely as ‘online risks’, but as harms with potentially serious psychological, social, and developmental consequences for young people.^18^

The manosphere describes a loosely connected online ecosystem of websites, forums, influencers, and communities that promote male supremacist and anti-feminist ideologies, often portraying women as manipulative, inferior, or responsible for men’s social and emotional difficulties.^13,19^ Emerging evidence suggests that social media recommender algorithms can amplify and normalise misogynistic narratives by repeatedly promoting related content to young users, increasing the visibility and reach of manosphere influencers and potentially shaping social norms around gender and relationships.^19^ As a result, girls and young women may encounter misogynistic material indirectly through mainstream social media environments, including memes, influencer culture, humour, gaming content, and recommendation systems, even when they are not actively seeking it out.^19,20^

Online sexual harassment is also evolving alongside technological change. Emerging forms include the creation of sexualised artificial intelligence (AI) imagery, where AI tools are used to generate non-consensual sexualised images of women as a means of humiliation, punishment, or silencing online.^21,22^ Recent evidence suggests that sexualised deepfakes are becoming increasingly visible within school contexts, raising concerns about psychological harm, reputational damage, and the normalisation of gender-based abuse through AI technologies.^22^ As these technologies become more widespread, their psychological and social impacts will require further investigation.^23^

For girls and young women, experiences of online misogyny are both common and increasing.^14,24,25^ The proportion of girls aged 13–21 reporting receiving sexist comments online has nearly tripled in a decade, rising from 20% in 2013 to 57% in 2023.^15^ In the 2025 Girls’ Attitude Survey by Girlguiding, 73% of girls aged 11-16 and nearly all young women (84%) aged 17-21 reported encountering sexism online.^26^ Social media recommendation systems are designed to maximise engagement and may progressively direct users towards increasingly extreme or polarising content, including misogynistic narratives associated with the manosphere.^13,19^ This algorithmic amplification can normalise hostile attitudes towards women and increase the likelihood that girls and young women encounter sexist or degrading content as part of everyday online experiences. Repeated exposure to these ‘everyday’ forms of misogynistic content may also make online misogyny difficult to identify, regulate, and measure within research.^20^ Qualitative studies suggest that online misogyny may negatively affect self-esteem, body image, emotional wellbeing, and perceptions of safety, while exposure to online sexual harassment and image-based abuse has been linked to anxiety, depression, shame, and suicidal ideation.^27–29^ Exposure to online sexual harassment may reinforce the threat of sexual violence and produce feelings of fear, helplessness and disempowerment which has negative implications for mental health.^30–32^ Research on technology-facilitated sexual harms further indicates that shame, embarrassment, fear of not being believed, and uncertainty about disclosure may intensify psychological distress among adolescents exposed to gendered online harms.^18^

Research and narratives on the mental health implications of online misogyny have focused largely on the increased risk of depression or other psychosocial difficulties in men and boys who engage with these communities, rather than the impact on girls and young women who are targeted by them.^33–38^ It is important to consider the role of gender when investigating online sexual harassment as most perpetrators are men, and most victims are women.^27,39–41^ Previous cross-sectional studies have reported a gendered negative impact of online sexual harassment on mental health, with girls and women experiencing more distress or harm.^41–45^ This has been attributed to online sexual harassment being perceived differently by girls and boys aged 16-24 years, whereby girls experience a more negative emotional impact, while boys downplay severity.^42,46,47^ Girls' and women’s experiences of online sexual harassment may be perceived as ‘an expression of gender hierarchies and patriarchal ideology’.^30,47–49^

Online misogyny is frequently encountered during adolescence, a formative stage of identity development in which beliefs about gender, relationships, and social norms are actively shaped.^19^ Emerging evidence suggests that misogynistic content is increasingly encountered through mainstream social media platforms rather than niche online communities alone, with algorithmic recommendation systems facilitating repeated exposure to gendered narratives during a sensitive developmental period.^19,20,50^ These experiences occur within a developmental window in which mental health difficulties, such as anxiety, depression, self-harm and eating disorders commonly begin in girls.^2^ As digital spaces play a central role in adolescent social lives, repeated or chronic exposure to hostile gendered content may have cumulative and lasting implications for gender attitudes and relationship expectations, as well as emotional wellbeing and psychological development.

Despite growing concern about online misogyny and its potential implications for adolescent wellbeing, important gaps remain in the evidence base.^15^ Existing research examining online misogyny has often been fragmented across related areas, including online sexual harassment, image-based abuse, pornography exposure, and manosphere content, rather than conceptualised as a broader gendered online harm. Much of the existing evidence linking online harms to mental health is also cross-sectional, limiting conclusions about temporality and directionality. Cross-sectional studies cannot determine whether exposure to online misogyny precedes declines in mental health, whether adolescents experiencing psychological distress are more likely to encounter or engage with misogynistic content, or whether these relationships are bidirectional. For example, depressive symptoms have been shown to predict subsequent exposure to sexualised content.^51^ Longitudinal evidence is therefore needed to clarify whether exposure to online misogyny represents a prospective risk factor for poor mental health outcomes.

To date, no systematic review has synthesised longitudinal evidence examining associations between exposure to online misogyny and subsequent mental health outcomes among girls, young women, and gender-diverse young people. To address this gap, the present systematic review synthesises longitudinal studies examining whether exposure to online misogyny is associated with later mental health outcomes among girls, young women, and gender-diverse young people (aged 11-25 years). The review examines different forms of online misogyny, including online sexual harassment, image-based abuse, misogynistic content exposure (including pornography), and related gender-based online harms, alongside outcomes including depression, anxiety, self-harm, eating disorders, body image concerns, and psychological wellbeing. The review also evaluates methodological limitations within the existing literature and identifies priorities for future longitudinal research.

Primary objective:

To establish whether exposure to online misogyny is longitudinally associated with mental health outcomes in girls, young women and gender-diverse people.

## Methods

This review was preregistered on the PROSPERO database (CRD420251125049).^52^ The PRISMA (Preferred Reporting Items for Systematic Reviews and Meta-Analyses) guidelines^53^ were followed (Supplementary file 1).

### Inclusion and exclusion criteria

Only longitudinal studies measuring mental health outcomes in young women, girls, gender-diverse people aged 11-25 years, following exposure to online misogyny were included. The protocol specified inclusion of studies involving young women and gender-diverse young people. However, no eligible studies involving gender-diverse participants were identified. The review therefore focuses on studies of girls and young women. Studies measuring exposure variables relating to online misogyny, including but not limited to i) gendered hate speech; ii) image-based sexual abuse (“revenge porn”); iii) trolling and sexual harassment; iv) exposure to misogynistic memes or subcultures (e.g. incel forums, manosphere) were included. Studies reporting any mental health outcome, including but not limited to depression, anxiety, self-harm thoughts and behaviours, eating disorder, self-esteem, body dissatisfaction, and psychological wellbeing were included. Studies were excluded if they: i) used cross-sectional or non-longitudinal designs; ii) examined misogyny or gender-based harms occurring offline only; iii) did not report mental health outcomes; iv) did not establish a temporal relationship between exposure and outcome; or v) included participants outside the specified age range without separate analyses for eligible participants. No restrictions were placed on date, geographic location, or language of publication. However, all studies meeting inclusion criteria were published in English. Full eligibility and exclusion criteria are presented in Supplementary file 2, Appendix 1.

To identify whether online misogyny may represent a potential causal risk factor for poor mental health, only studies that establish a temporal relationship were included, addressing concerns about reverse causality. Restricting to longitudinal design was also intended to improve the quality of evidence.

### Identification of studies

A comprehensive literature search was conducted in PsycINFO (Ovid), Web of Science, PubMed and Europe PMC, 4^th^ September 2025. Search terms combined concepts relating to: i) online misogyny and gender-based online harms (e.g. misogyny, sexual harassment, sexism, “revenge porn”, incel, manosphere); ii) digital environments (e.g. online, social media, internet, gaming); iii) mental health outcomes (e.g. depression, anxiety, psychological distress, self-harm, wellbeing); and iv) longitudinal study designs (e.g. cohort, prospective, follow-up, panel studies). Controlled vocabulary terms (e.g., MeSH terms) and free-text terms were adapted for each database. Full search strategies for all databases are provided in Supplementary File 3. Records were processed, stored and screened using Rayyan.^54^ Two authors (LW and CT) independently (and blinded) screened all the papers for inclusion based on the title and abstract simultaneously, with disagreements resolved by discussion. The same two authors independently reviewed the full text papers for inclusion, again, with disagreements also resolved by discussion. The title-abstract screening had a concordance of 94%, and 100% concordance after discussion and conflict resolution. Full text screening had a concordance of 89% and 100% after discussion and conflict resolution.

Descriptive data on the key characteristics of each study were extracted into an Excel database. Extracted data included sample size and demographic information, how exposure to online misogyny was defined and operationalised, mental health outcomes and measurement, and key findings reported (full list available in Supplementary file 2, Appendix 2).

To conduct a meta-analysis, at least two of the studies included in this review had to assess the same exposure with the same measure, use the same outcome with the same measure, and report the same measure of effect. As none of the studies met the criteria it was not possible to perform meta-analysis. Therefore, a narrative synthesis was conducted to describe and summarise the characteristics and results of the included studies.

Risk of bias was assessed using the Newcastle-Ottawa Scale.^55^ Two authors (LW and CT) independently (and blinded) assessed all included studies for risk of bias, with disagreements resolved by discussion.

## Results

In total, 156 studies were identified through database searching and no additional studies were identified through handsearching. 137 were excluded after title and abstract screening (Figure 1). Of the 19 studies included in full-text screening, six studies met inclusion criteria, reflecting a small and methodologically diverse body of longitudinal research examining associations between online pornography exposure or cyber-sexual harassment and later mental health outcomes. The mean age of participants in included studies ranged from 12.43 to 21.06 years. The total number of participants (total N=7,379 across all studies) ranged from 143^56^ to 3,064.^57^

**Figure 1.**
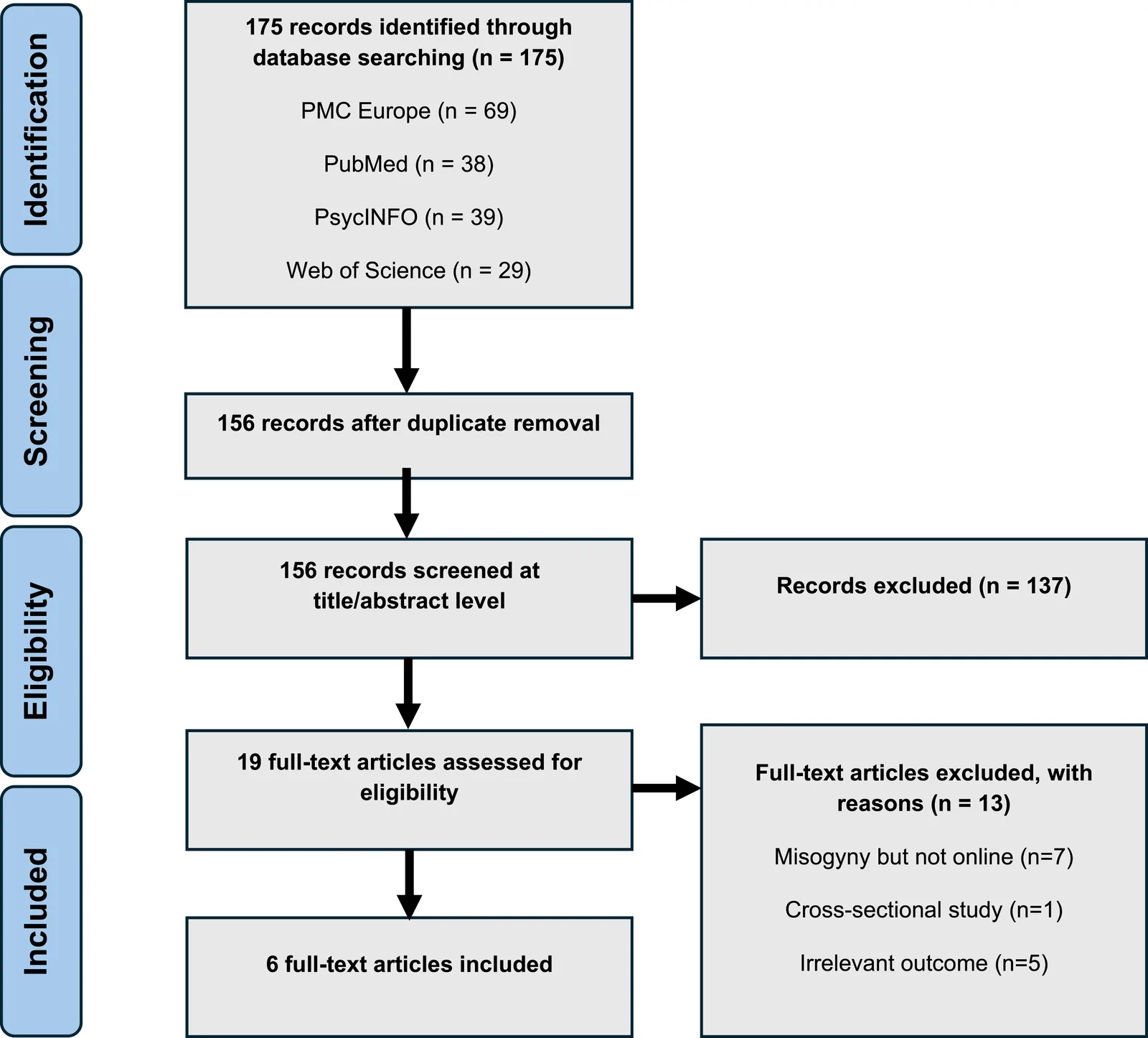
PRISMA diagram of included studies from literature search on 4^th^ September 2025.

The included studies were conducted in the USA (n = 2), Taiwan, Canada, Hong Kong and the UK (all n = 1). Four studies reported participant ethnicity and had predominantly White populations.^56–59^ Two studies reported participant sexuality and had predominantly heterosexual populations.^56,59^ Four studies had a mixed sex/gender group of participants, and two were single gender.^56,59^ Two of the mixed sex/gender studies were mostly sex/gender balanced,^57,58,60^ although one study included fewer than 40% females.^61^ No studies reported recruiting any gender-diverse young people. A summary of the included studies is presented in Table 1.

**Table 1.** Characteristics, aims and key findings of the studies included in the systematic review.

Authors, year, country	Journal	Population (mean age (range), sex/gender (% female/women/girls), demographics, setting)	Follow- up	Sample size	Exposure	Outcome	Effect size (Female-specific results and gender analysis if reported)	Key findings No evidence Risk factor Protective factor	Newcastle-Ottawa quality assessment
Chao & Yu (2023) Taiwan	Int. J. Ment. Health Addict.	Baseline: 16.99 Follow-up: 18.49 High school students Sex: Baseline: Females N=334 Follow-up: Females N=318	2 years	1838	Cyber pornography	Physical and mental health	Females: Regression coefficient: −0.062 Males: Regression coefficient: −0.019 Gender difference: t=0.042	Cyber pornography had a non-significant effect on physical and mental health 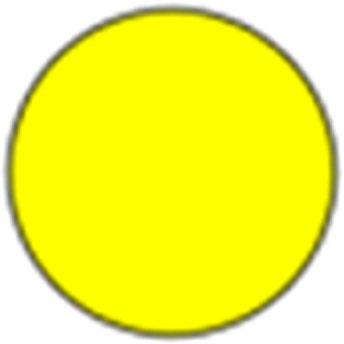	Fair
					Cyber pornography x self-consciousness		Regression coefficient: 0.025*		
					Cyber pornography x self-consciousness x peer relationships		Regression coefficient: 0.024**		
					Cyber pornography x peer relationships		Regression coefficient: 0.068**		
Copp et al. (2021) USA	J. Adolesc.	Wave 1: 13.57 (10-18), 48.78% Female; nationally representative panel Wave 1: Household income above 2013 median: 53.04%; Parental education less than high school: 7.47%, Some college: 19.88%, College or more: 46.44%; Two-parent household: 78.30% Wave 1: Black: 8.77%, Hispanic: 17.80%, Other: 6.99%	2 years (wave 2 and 4 used from the National Survey on Teen Relationships and Intimate Violence (STRiV))	1152	Online sexual harassment victimization at wave 2	Depressive symptoms at wave 4	Females Regression coefficient: 0.938 (OR = 2.55***) for Males Regression coefficient: 0.137 (OR = 1.15)	The effect of online sexual harassment on depressive symptoms was stronger among female respondents 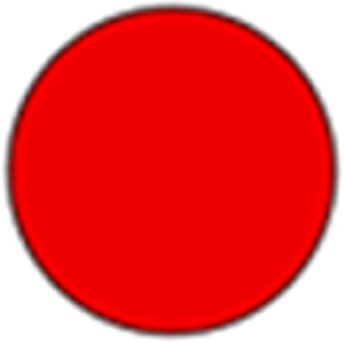	Fair
Daskaluk (2022) Canada	Unpublished dissertation	20.1 (18-25), 100% Female University students Ethnicity: 40.3% European; 11.2% Asian; 7.6% Black/African; 2.9% Hispanic; 2.4% Indigenous; 1.2% North African; 33.5% Multiethnicity Sexuality: Heterosexual 88.2%; 9.1% bisexual; 1.2% gay/lesbian; 1.2% pansexual; 0.3% demisexual	4-months	Baseline: 329 Follow-up:143	Cyber sexual aggression	Self-esteem	Correlation coefficient: -0.20* Standardized indirect effect of Cyber sexual aggression at Time 1 to self-esteem at Time 2: Regression coefficient = -0.174**	CSA victimization at baseline was significantly indirectly negatively associated with self-esteem and DASS at follow-up. 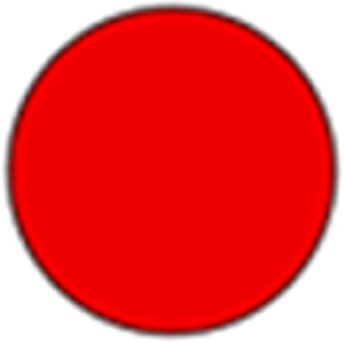	Fair
						Depression, anxiety and stress	Correlation coefficient: -0.26*** Standardized indirect effect of Cyber sexual aggression at Time 1 to DASS at Time 2: Regression coefficient = -0.182**		
Dollimore et al. (2025) UK	Psychol. Pop. Media	21.06 (18-25), 100% young women (University students) Ethnicity: White British = 72%; Asian = 9%; mixed = 4.8%; Black = 4.5%; Arab = 1%; Other = 8% Sexuality: Heterosexual = 73.2%; bisexual = 17.2%; gay/lesbian = 2.9%; other = 3%; unsure = 2.5%; prefer not to say = 1%	10-weeks	Baseline: 314; Follow-up: 207	Sexual harassment on social media	Body shame	T1 sexual harassment -> T2 body shame: not measured directly	Time 1 sexual harassment was not associated with changes in self- objectification, body shame, and personal safety anxiety 10 weeks late 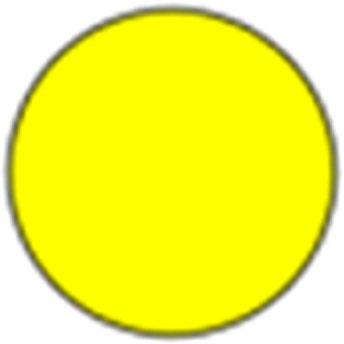	Poor
						Personal Safety Anxiety	T1 sexual harassment -> T2 self-objectification: Regression coefficient =0.01; T2 self-objectification -> T2 physical safety anxiety: Regression coefficient =0.00		
						Self-Objectification	Non-significant		
Lawrence et al., (2025) USA	Child Psychiatry Hum. Dev.	Wave 1: 14.771 Wave 2: 15.282 Wave 3: 16.672 Four high schools in Midwestern USA Wave 1: 50.34% Female Wave 1: 26.21% Black (N = 803), 47.85% White (N = 1466), 20.40% Latinx (N = 625), 2.19% Asian (N = 67), and 3.36% Biracial (N = 103).	Three wave study - Autumn 2009, Spring 2012, Autumn 2012)	3064	Cyber-sexual harassment	Anxiety and depressive symptoms	*Female-specific results not available (full sample only) Risk of assignment to depression/anxiety symptoms trajectory groups High: Reference Moderate: − 0.552*** Low: − 1.603*** Declining: − 0.801	Baseline cyber sexual harassment victimization positively predicted the High Chronic class as consistent with the correlated liability model of psychopathology. 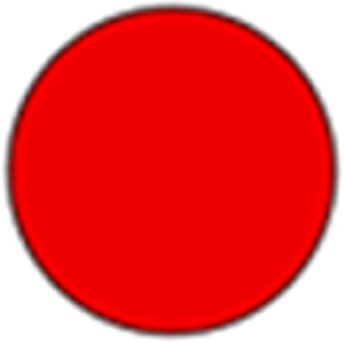	Fair
Ma et al. (2019) Hong Kong	Applied Research in Quality of Life	Wave 1: 12.43 (NR), 46% females Students in 13 secondary schools	Two 12- month follow-ups	1091	Intentional and non-intentional exposure to online pornography	Depression	*Female-specific results not available (full sample only) Intentional exposure to online pornography Regression coefficient: 0.19** Non-intentional exposure to online pornography: non-significant	Adolescents exposure to online pornography was associated with depressive symptoms. Intentional exposure appears to be associated with negative psychological wellbeing. A higher level of exposure to online pornography is associated with depressive symptoms and lower levels of life satisfaction. 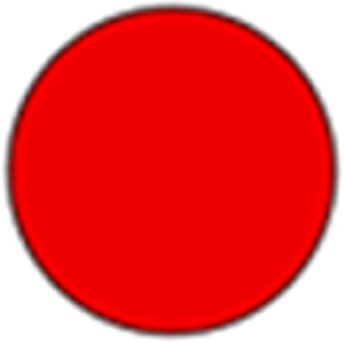	Fair
						Life Satisfaction	*Female-specific results not available (full sample only) Intentional exposure to online pornography: Regression coefficient: −0.14** Non-intentional exposure to online Regression coefficient: −0.12**		

Statistical significance levels are indicated by the asterisks: *p<0.05 **p<0.01 ***p<0.001.

The studies included in this review often had an inconsistent approach to ‘sex’ and ‘gender’ terminology. Sex is a biological characteristic i.e. ‘male or female’,^62^ whereas ‘gender’ refers to socially constructed norms, roles and expectations of women, men, girls and boys.^63,64^ The terms ‘male’ and ‘female’ are sex-specific terminology and ‘woman’, ‘man’, ‘girl’, and ‘boy’ are gender-specific.^65^ Two studies followed this terminology consistently: pairing ‘gender’ with ‘women’.^56,59^ The four remaining studies explored the role of ‘gender’ but used the binary sex-specific ‘male and female’ approach to analyses.^57,58,60,61^ This review uses the same sex-specific or gender-specific terminology used by the authors; when studies use only gender-specific terminology this review does the same. However, when studies use sex and gender-specific terminology interchangeably this review uses sex-specific terminology ‘male’ and ‘female’.

Methodological approaches varied substantially across studies. Across the review, study definitions of online misogyny were heterogenous, ranging from online sexual harassment and technology-based aggression to pornography exposure, and measures captured different combinations of behaviours, including sexualised comments or jokes, spreading sexual rumours, image-based abuse, coercive messaging, and humiliation. Exposures (Table 2) were operationalised using a range of measures, including online sexual harassment victimization,^58^ Cyber Sexual Aggression,^56^ cyber sexual harassment,^57^ study-specific measures of sexual harassment on social media,^59^ and cyber/online pornography.^60,61^ Mental health outcomes (Table 2) were similarly heterogeneous and included depressive symptoms^58,60^ (e.g. MHI-5,^66^ PHQ-9^67^), anxiety and stress^56^ measured using the Depression Anxiety Stress Scale (DASS^68^), a combined measure anxiety and depressive symptoms,^57^ self-esteem^56^ measured using the Rosenberg Self-Esteem Scale,^68^ self-objectification and body shame,^59^ (Objectified Body Consciousness Scale^69^), the Personal Safety Anxiety and Vigilance Scale,^59,70^ and the Satisfaction with Life Scale.^60,71^ Follow-up periods ranged from 10 weeks^59^ to two years.^57,58,61^ Analytic approaches included regression modelling,^56,58–61^ latent trajectory analysis,^57^ mediation analysis,^59^ correlations,^56^ and odds-ratio estimation,^58^ with varying levels of adjustment for baseline mental health and demographic confounders. This methodological heterogeneity limited comparability between studies and precluded meta-analysis.

**Table 2.** Exposure and outcome measures.

Authors, year, country	Exposure	Exposure measure	Outcome	Outcome measure
Chao & Yu (2023) Taiwan	Cyber pornography	Questionnaires adapted from previous studies.^72–78^ Items scored with a 5-point Likert scale from 1 (strongly disagree) to 5 (strongly agree). Questionnaire item wording not provided.	Physical and mental health	Questionnaire: Four items adapted from previous studies.^72,75,78^ Items scored with a 5-point Likert scale from 1 (strongly disagree) to 5 (strongly agree). Questionnaire item wording not provided.
	Cyber pornography x self-consciousness			
	Cyber pornography x self-consciousness x peer relationships			
	Cyber pornography x peer relationships			
Copp et al. (2021) USA	Online sexual harassment victimization at wave 2	Questionnaire: Respondents asked how often anyone had done the following actions to them online the past year: 1) Made unwelcome sexual comments, jokes, or gestures 2) Spread unwelcome sexual rumours 3) Called you gay or lesbian in a negative way 4) Tried to get you to talk about sex 5) Asked you for sexual info about yourself when you did not want to tell the person 6) Asked you to do something sexual when you did not want to. Items were score 1 “never” to 3 “more than once”. Responses were dichotomized to create a single binary variable for any online sexual harassment victimization (1 = yes).	Depressive Symptoms at wave 4	Mental Health Inventory (MHI-5)^66^ was developed from the Short Form 36 (SF-36) questionnaire to assess depressive symptoms (e.g. feeling particularly nervous, downhearted and blue).^79^ Item scores were summed and transformed with standard linear transformation. Scores were dichotomised by coding a score of less than 77 as 1, indicating a screen for a “common mental disorder”.
Daskaluk (2022) Canada	Cyber sexual aggression (CSA)	Cyber Sexual Aggression questionnaire developed from five previous questionnaires: 1) The modified Sexual Experiences Questionnaire: 15 items e.g. “has continued to ask you for dates, drinks, dinner, etc., even though you have said no”^80^ 2) Sexual cyber abuse scale: four victimization items e.g. “pressured you to send a sexual or naked photo of yourself”^81^ 3) The modified Lifetime Cybersex Experience Questionnaire: two items e.g. “described their sexual fantasies and/or sexual desires to you that made you uncomfortable”^82^ 4) The modified Technology-Facilitated Sexual Violence Questionnaire: 10 items (nine original items e.g. “have you been virtually raped [i.e., having your avatar or digital representation of yourself subjected to sexual violence by other avatars]” and one new item “sent you, unknowingly and without your consent, to webpages that feature graphic and sexual images)^83^ 5) Items for online sexual harassment: (e.g. “made unwanted comments or asked about your appearance or your body”)^84^ Time 1: Respondents asked to report experiences of CSA victimization during past year on 9-point Likert-type scale from 0 (never) to 8 (5 or more times a day). Time 2: Respondents asked to report experiences of CSA victimization since Time 1 (last four months) on a 7-point Likert-type scale from 0 (never) to 6 (5 or more times a day).	Self-esteem	The Rosenberg Self-Esteem Scale^68^ Ten 4-point Likert-type questions (e.g. “on the whole, I am satisfied with myself”). Items scored from 1 (strongly disagree) to 4 (strongly agree). Higher scores represented higher self-esteem.
			Depression, anxiety and stress	The Depression Anxiety and Stress Scale (DASS).^68^ Twenty-one 4-point Likert questions (e.g., “I couldn’t seem to experience any positive feeling at all”), scored from 4 (Applied to me very much, or most of the time) to 1 (did not apply to me at all). Scores were reversed for higher scores to indicate lower levels of depression, anxiety, and stress (matching the self-esteem measure)
Dollimore et al. (2025) UK	Sexual harassment on social media	Eight item ‘sexual harassment on social media’ scale developed from previous questionnaire^80^ to capture women’s experiences of everyday harassment on social media and experiences of unwanted sexual advances. Five-point Likert scale ranging from 1 (never) to 5 (very often).	Body shame	Objectified Body Consciousness Scale OBCS body shame sub-scale.^69^ Each item in this scale was also rated on a 5-point Likert scale from 1 (strongly dis-agree) to 5 (strongly agree).
			Personal Safety Anxiety	Personal Safety Anxiety and Vigilance Scale.^70^ Eight items rated on a 7-point Likert scale from 1 (completely unlike me) to 5 (completely like me).
			Self-Objectification	Objectified Body Consciousness Scale Body surveillance subscale.^69^ Each item rated on a 5-point Likert scale from 1 (strongly disagree) to 5 (strongly agree).
Lawrence et al., (2025) USA	Cyber-sexual harassment	Three items adapted from the Technology Aggression Victimization scale^85^ measuring cyber sexual harassment victimization in the past year. 1) “Someone online asked me for sexual information about myself that I didn’t want to tell them” 2) “Someone tried to get me to talk about sex online when I didn’t want to” 3) “Someone asked me to do something sexual online that I did not want to do.” Items scored using a 5-point Likert scale from 0 (Never) to 4 (Seven or more times). Higher scores indicated more frequent cyber sexual harassment.	Anxiety and depressive symptoms	Orpinas Modified Depression Scale^86^ measuring the extent of anxiety and depressive symptoms in the last 30 days, items scored using a 5-point Likert scale ranging from 0 (Never) to 4 (Always).
Ma et al. (2019) Hong Kong	Intentional and non-intentional exposure to online pornography	Six item questionnaire on respondents’ exposure to online pornography and the frequency of intentional exposure to a) online pornographic stories, b) pornographic pictures, c) pornographic videos, d) pornographic advertisement, e) pornographic websites, and f) online sex chat “How often you were intentionally exposed to online pornographic stories over the past six months?” The same questions were asked for non-intentional exposure. Items were scored 0 (never), 1 (at least once a month), 2 (1–3 times a month), 3 (at least once a week), 4 (several times a week) and 5 (everyday). The six items were averaged to create a mean score, with higher scores indicating higher exposure to online pornography.	Depression	Patient Heath Questionnaire (PHQ-9)^67^ was used to measure depressive symptoms on a four-point scale. Items scored 0 (not at all), 1 (several days), 2 (more than half the days), 3 (nearly every day). A mean score was calculated. Higher mean scores indicated higher depressive symptoms (e.g. feeling tired or having little energy).
			Life Satisfaction	Satisfaction with Life Scale.^71^ Items were rated on a 6-point scale. Items were scored form 1 (strongly disagree) to 6 (strongly agree). A higher score indicated more life satisfaction.

Evidence linking online misogyny exposures to later mental health outcomes was mixed. Studies from the USA and Taiwan suggested that online sexual harassment victimisation represents a mental health risk factor, particularly for girls: Copp et al. (2021) reported stronger increases in depressive symptoms among female adolescent respondents compared with males at two-year follow-up, distinguishing online sexual harassment from cyberbullying.^58^ There was evidence of compounding harm, where young people who experienced both cyberbullying and online sexual harassment had significantly higher odds of later depression and poorer wellbeing than those experiencing only one. Chao and Yu (2023) found that baseline cyber-pornography exposure predicted poorer peer relationships at two-year follow-up, which in turn related to wellbeing, although the overall regression effect on combined physical and mental health was non-significant.^61^ Complementary evidence from Canada supported this pattern, with Daskaluk’s (2022) longitudinal study of cyber-sexual aggression showing that victimisation at baseline was negatively correlated with later self-esteem and Depression Anxiety and Stress (DASS) scores.^56^ Significant indirect effects of Cyber sexual aggression (CSA) on mental health at four-month follow-up suggested that experiencing aggression may compound existing mental health vulnerabilities.

Further longitudinal studies that included depression and anxiety as outcomes were available only for the full samples rather than for females alone. Lawrence et al. (2025) analysed a three-wave US cohort, finding that cyber-sexual harassment at baseline predicted membership of a ‘high-chronic internalising’ trajectory class (though gender-specific results were not available), experiencing persistently elevated anxiety and depression over time.^57^ Ma (2019) reported that intentional, though not incidental, pornography exposure was associated with depressive symptoms (PHQ-9) and lower life satisfaction in early Hong Kong adolescents, though it was unclear whether these non-sex/gender-specific findings were observable in the female-only sample.^60^

Dollimore et al.’s (2025) research in young women studying at a UK university, using a bespoke eight-item ‘sexual harassment on social media’ scale, found cross-sectional associations with body shame and self-objectification but no evidence of associations at ten-week follow-up, suggesting that short-term impacts may be limited or require longer periods to manifest.^59^

Overall, the available evidence provides preliminary support for online sexual harassment and related forms of online misogyny as potential longitudinal risk factors for poorer mental health among adolescent girls and young women. However, substantial heterogeneity in exposure definitions, outcome measures, follow-up periods, and analytic approaches limits comparability across studies and precludes firm conclusions.

### Quality of studies

Newcastle-Ottawa assessment indicated the six included studies were generally of moderate (fair) methodological quality, with the exception of Dollimore et al., (2025) which was rated poor.^59^ Most studies relied on written self-report to ascertain exposure and only one study used a population-representative cohort.^58^ Two studies did not control for baseline mental health or exclude baseline cases, limiting causal inference. In the comparability domain, five studies controlled for a range of confounders through design or analysis, while Dollimore et al. (2025) did not report controlling for any demographic variables.^59^ All studies used self-reported outcome measures, and follow-up durations varied widely, from 10 weeks to two years, with Dollimore et al. (2025) judged too short for outcomes to be observed.^59^ Loss to follow-up exceeded 20% in five studies without adequate description of attrition.^56,58–61^ This contributes to risk of bias and underpinning the overall classification of evidence as predominantly fair but not robust. A summary of the quality assessment is presented in Supplementary file 2, Appendix 3.

### Certainty of evidence

A Grading of Recommendation, Assessment, Development and Evaluation (GRADE) was used to rate the certainty of evidence.^87^ The overall rating of the certainty was ‘moderate’ due to risk of bias and inconsistency. A summary of the GRADE assessment is presented in Supplementary file 2, Appendix 4.

## Discussion

This review highlights a substantial gap in the longitudinal evidence base examining how online misogyny shapes mental health outcomes in girls, young women and gender-diverse young people. Digital environments are now central to young people’s social and emotional development. However, empirical research in this area remains scarce, heterogeneous, and largely descriptive. Online misogyny is thus under-represented within developmental mental health research, policy, and prevention frameworks, despite its prevalence and potential harm.

### Summary of evidence

We identified six studies that tested longitudinal associations between exposure to online misogyny and subsequent mental health outcomes. None were assessed as high quality, with one study assessed to be limited by short follow-up, high attrition and analytic constraints. Definitions of online misogyny varied considerably, with four studies operationalising exposure as online sexual harassment and two focusing on exposure to online pornography. Even within these categories, measures were inconsistent, capturing different combinations of behaviours and experiences. Outcomes and analytic approaches were similarly heterogeneous, limiting comparability and precluding quantitative synthesis.

Despite these limitations, the available evidence provides preliminary support for prospective associations between exposure to online misogyny and poorer mental health. Of the four studies reporting sex/gender-specific results, two identified negative longitudinal associations between online sexual harassment and mental health outcomes.^56,58^ Two studies reported combined results only.^57,60^ Dollimore et al. (2025) observed cross-sectional but not longitudinal associations between online sexual harassment and body shame and self-objectification,^59^ while Chao et al. (2023) found no direct longitudinal association between cyberpornography exposure and later mental health.^61^ Taken together, these mixed findings suggest that effects may vary by type of exposure, outcome, developmental timing, and analytic approach, rather than indicating an absence of harm.

These findings align with robust evidence from offline contexts. Longitudinal research on sex discrimination and misogyny in everyday life demonstrates consistent associations with psychological distress, poorer mental functioning, and reduced life satisfaction among women.^88–90^ The consistency between existing offline evidence and this synthesis of the limited literature on online misogyny strengthens the plausibility of this as a gendered psychosocial stressor, even though direct longitudinal evidence remains limited.

### Critical gaps and conceptual limitations

A striking finding is the absence of longitudinal research focused explicitly on gender-diverse or LGBTQ+ young people, despite evidence that these groups experience disproportionate exposure to online harassment and misogyny.^91^ None of the included studies examined experiences of exposure to explicitly misogynistic online subcultures, such as the manosphere, nor did any compare the relative mental health impacts of online versus offline misogyny. Only one study^58^ attempted to disentangle online sexual harassment from broader cybervictimisation, limiting comparisons between the mental health impact of cybervictimisation and misogyny-specific effects.

The review also reveals a narrow operationalisation of online misogyny. Only two forms (online sexual harassment and pornography exposure) were represented. While both are relevant, they capture only a fraction of how misogyny is enacted and reinforced online. Online sexual harassment is commonly gendered, and can form part of wider cultures of misogyny in digital spaces.^14,19^ Similarly, mainstream online pornography often depicts gender roles, bodies, and sexual practices in ways that reinforce harmful beliefs about women, consent, and sexual entitlement, including humiliation, coercion, and aggression. When pornography serves as a primary source of sexual learning, these portrayals may contribute to internalised misogyny and distorted expectations of gendered relationships.^92^ However, misogyny also operates through routine, indirect and chronic exposure to sexist content, algorithmically amplified narratives, influencer cultures, and hostile discourse that may not involve direct victimisation but nonetheless shape identity, self-worth, and perceived social norms.^13,17,19^ Measuring the impact of this non-targeted, pervasive or incidental exposure to misogynistic content is identified as a critical evidence gap given its widespread prevalence among girls and young women.^27–29^ This review underlines that finding.

The absence of intersectional analysis constitutes a further major limitation. Experiences of online misogyny are not uniform; they are structured by race, sexuality, gender identity, and disability. Intersectionality theory emphasises that discrimination operates through mutually reinforcing systems of oppression rather than isolated categories.^93,94^ For example, Black women frequently experience ‘misogynoir’, gendered racism that combines anti-Blackness and misogyny in distinct and intensified forms (both online and offline).^95,96^ Similarly, LGBTQ+ young people may experience misogyny intertwined with homophobia and transphobia, producing qualitatively different forms of hostility and psychological impact.^97^ The studies included in this review were not designed to disentangle these layered experiences, and none examined how intersecting identities might modify associations between online misogyny and mental health. As a result, the evidence base risks obscuring how structural inequalities compound exposure and harm. Future longitudinal research should therefore adopt intersectional frameworks, measure multiple overlapping forms of digital discrimination (e.g., gendered racism, homophobic and transphobic harassment), and examine whether mental health impacts differ across intersecting identities.

### Mental health outcomes and mechanisms

Evidence gaps are particularly notable in relation to specific mental health outcomes and mechanisms. Few studies examined self-esteem, depression or anxiety, and none tested online misogyny as a longitudinal predictor of self-harm, suicidal ideation, or disordered eating, despite strong theoretical links. Misogynistic online environments frequently involve sexualisation, evaluation, and surveillance of girls’ bodies, processes theorised to promote self-objectification and body dissatisfaction. Yet only one study examined associations with self-objectification longitudinally.^59^

Future research should explicitly test mechanistic pathways, including: internalised gendered stigma influencing self-esteem and depression^98^; body surveillance and appearance anxiety contributing to eating pathology^99^; repeated exposure to hostility increasing stress and anxiety^100^; and social rejection or exclusion leading to loneliness and internalising symptoms.^101,102^ For experiences such as image-based abuse, plausible pathways include loss of trust, self-blame, trauma symptoms, and depression or anxiety.^103^

Follow-up periods in the included studies were highly variable (10-weeks to two-years), and more insight is required into longer-term developmental risk. While findings suggest associations with medium-term distress, no studies examined whether exposure to online misogyny contributes to longer mental health trajectories across adolescence and into young adulthood.

### Measurement and methodological challenges

The review underscores the need to distinguish between direct, targeted misogynistic victimisation (e.g. receiving sexualised messages or threats) and ‘routine’ exposure to misogynistic content (e.g. sexist videos, manosphere narratives, hostile commentary). These experiences are likely to differ in intensity, mechanism, and mental health impact, yet remain poorly differentiated in existing measures. More broadly, misogyny often operates implicitly or anticipatorily; anticipated discrimination can be psychologically harmful even in the absence of discrete events, posing challenges for measurement reliant on recall of specific incidents.^104^ Advancing the field will require new, context-sensitive measurement approaches that capture exposure to misogynistic content beyond screen time or generic harassment metrics.

### Strengths and limitations

A key strength of this review is its focus on longitudinal evidence, allowing assessment of temporal ordering that cross-sectional studies cannot provide. While this narrow inclusion criteria limited the number of eligible studies, it was essential for addressing questions of developmental risk. Cross-sectional research can demonstrate co-occurrence but cannot establish whether exposure precedes distress, whether distressed individuals are more likely to encounter misogyny, or whether bidirectional processes operate. As with other mental health risk factors, such as cyberbullying and face-to-face bullying, disentangling online from offline misogyny remains challenging, given their close interrelation in everyday life.^105,106^ As digital and offline social worlds become increasingly interconnected, distinctions between online and offline gender-based harm are becoming increasingly blurred.^18^ Online misogyny may therefore reflect, reinforce, and amplify broader patterns of gender inequality and violence experienced by girls and women offline. The studies included in this review were not clear about how the ‘sex’ or ‘gender’ of participants was obtained and sex/gender terms were used interchangeability. This introduces a risk of misgendering participants.^63^

Web of Science was selected as the primary multidisciplinary citation database. We did not additionally search Scopus due to the substantial overlap in indexed journals but it is possible that some eligible papers could have been missed.

A further limitation relates to the broader structural framing of online misogyny. While this review primarily discusses misogyny in relation to patriarchal gender norms and inequalities, online harms are also shaped by intersecting systems of power, including racism, colonialism, heteronormativity, ableism, and socioeconomic inequality. These systems may influence both the forms online misogyny takes and the ways harms are experienced across different groups and cultural contexts. However, the limited evidence base identified in this review did not permit detailed examination of these broader structural processes. Future research and evidence syntheses could adopt more intersectional and globally informed frameworks for understanding how overlapping systems of oppression shape digital experiences and mental health outcomes.

### Broader context and future directions

The studies included in this review were conducted across several countries, but little is known about how online misogyny may differ across cultural and social contexts. Most studies were conducted in high-income Western countries, with a smaller number from East Asian settings including Taiwan and Hong Kong. No eligible longitudinal studies were identified from low- and middle-income regions such as Africa, Latin America, or South Asia. This is an important gap given the rapid global growth of digital technology and social media use among adolescents.^18^ The lack of research from many parts of the world may reflect wider inequalities in mental health and digital research, including differences in funding, access to longitudinal data, and publication visibility.^107^ Future research should include more culturally and geographically diverse populations to better understand how online misogyny affects young people across different social and technological contexts.

Rapid technological change further complicates this landscape, with emerging forms of misogyny occurring through gaming platforms,^108^ avatar-based harassment, and AI-generated sexualised or degrading representations of women and girls.^21,22^ Recent research highlights the growing use of sexualised deepfakes within school settings, where AI-generated images are used to harass, humiliate, and exert power over girls, often in ways that existing safeguarding systems are poorly equipped to address.^22^ These developments suggest that online misogyny is evolving beyond text-based harassment and image-sharing practices toward increasingly sophisticated forms of technology-facilitated sexual violence. Longitudinal research will need to keep pace with these changes and examine whether emerging AI-enabled forms of abuse have distinct psychological consequences for young people. Longitudinal designs – using established cohort studies where available – should further explore developmental sensitivity and examine differential impacts for groups disproportionately targeted online, including girls of colour, LGBTQ+ youth, neurodivergent young people, and those facing socioeconomic disadvantage.^109^

Our review supports growing calls to move beyond broad or exposure-based measures of digital technology use and instead examine how specific forms of online harm shape adolescent wellbeing over time.^18^ Online misogyny may be better conceptualised as a gendered social and developmental exposure embedded within young people’s everyday digital environments, rather than as an isolated online ‘risk’. Online misogyny should be understood as part of the broader social determinants of girls’ and women’s mental health, reflecting and reinforcing offline gender norms, power relations, and inequalities. Recognising this has implications for policy, platform design and regulation, online safety and media literacy education, and gender-sensitive mental health prevention and support strategies. The lack of scientifically rigorous research examining exposure to online misogyny as a potential predictor of poor mental health is particularly notable against the backdrop of high numbers of girls, young women and gender-diverse people globally encountering misogynistic content and interactions online. This population is routinely exposed to online sexual harassment, body shaming, image-based abuse, and misogynistic peer cultures widespread.^24–26^ Despite this, these experiences remain peripheral in mental health research, policy, and clinical practice, reflecting enduring patriarchal structures. As a result, the scale and nature of harm is likely underestimated, limiting the prioritisation and development of interventions beyond self-regulation approaches.^36^

### Clinical, prevention, and policy implications

The findings of this review have important implications for clinical practice, prevention, and public mental health policy. Clinicians working with adolescent girls and gender-diverse young people should consider exposure to online misogyny as a potential psychosocial stressor. Routine clinical assessments should capture gendered digital experiences, as these exposures may compound existing vulnerabilities and contribute to distress. Preventive approaches in schools and community settings should move beyond generic online safety messaging to explicitly address gendered harms, misogynistic norms, and algorithmically amplified content, integrating these issues within mental health and suicide prevention strategies. Interventions that strengthen critical digital literacy, challenge internalised gender stigma, and support emotion regulation and help-seeking may be particularly beneficial.^110^

At a policy and systems level, the limited longitudinal evidence identified in this review should not be interpreted as evidence of absence of harm, but rather as a failure of the research agenda to keep pace with young people’s digital realities. Given the scale of exposure to online misogyny globally, there is an urgent need for longitudinal, mechanism-focused research that treats online misogyny as a social determinant of mental health. Such work is essential to inform gender-sensitive clinical guidance, targeted prevention efforts, and platform regulation aimed at reducing psychological harm.

## Conclusions

This review synthesises the limited longitudinal evidence on the mental health implications of online misogyny for girls, young women and gender-diverse young people and highlights substantial gaps in the current knowledge base. Despite the importance of digital environments to young people’s social and emotional development, research examining online misogyny as a predictor of mental health outcomes remains scarce, methodologically heterogeneous, and narrowly operationalised. As a result, the potentially enduring impact of gendered online harms on mental health is likely underestimated.

Longitudinal, theory-driven research that captures diverse forms of misogyny, accounts for intersecting inequalities, and tests mechanistic pathways is urgently needed. Without this evidence, prevention, clinical practice, and policy responses will continue to lag behind the realities of young people’s digital lives. Recognising online misogyny as a public mental health issue is a necessary step toward developing more effective, gender-sensitive strategies to protect and promote mental health across adolescences.^106^

## Supplemental Material

Supplemental Material - Is exposure to online misogyny associated with mental health outcomes in girls and young women? A systematic review of longitudinal studies Supplemental Material for Is exposure to online misogyny associated with mental health outcomes in girls and young women? A systematic review of longitudinal studies by Ciara Thomas, Laura Tinner, Jacks Bennett, Lucy G Anderson, Lizzy Winstone in Women's Health.

Supplemental Material - Is exposure to online misogyny associated with mental health outcomes in girls and young women? A systematic review of longitudinal studies Supplemental Material for Is exposure to online misogyny associated with mental health outcomes in girls and young women? A systematic review of longitudinal studies by Ciara Thomas, Laura E Tinner, Jacks Bennett, Lucy G Anderson, Lizzy Winstone in Women's Health.

Supplemental Material - Is exposure to online misogyny associated with mental health outcomes in girls and young women? A systematic review of longitudinal studies Supplemental Material for Is exposure to online misogyny associated with mental health outcomes in girls and young women? A systematic review of longitudinal studies by Ciara Thomas, Laura E Tinner, Jacks Bennett, Lucy G Anderson, Lizzy Winstone in Women's Health.

## Data Availability

All data analysed during this study are included in this published article and its supplementary material. The full search strategies supporting the findings of this study are available in Supplementary file 3.*
