# Supplementary material for: Is exposure to online misogyny associated with mental health outcomes in girls and young women? A systematic review of longitudinal studies
Source: Womens Health (Lond). 2026 Sep 23;22:17455057261485065. doi: 10.1177/17455057261485065 (PMC13735633; doi:10.1177/17455057261485065)
Supplement: Supplementary file 2 [file sj-pdf-2-whe-10.1177_17455057261485065.pdf]

Is exposure to online misogyny associated with mental health outcomes in girls and young women? A systematic review of longitudinal studies

Supplementary file 2. Appendices

Appendix 1. Eligibility criteria

Criteria	Inclusion	Exclusion
**Study design**	Longitudinal study design	Cross-sectional design
**Population**	Young women, girls, gender-diverse people aged 11-25 years.	Studies with outcomes only measured in male participants.
**Exposure**	Studies measuring exposures variables relating to online misogyny, including but not limited to:<br>– Gendered hate speech<br>– Image-based sexual abuse ("revenge porn")<br>– Trolling and sexual harassment<br>– Exposure to misogynistic memes or subcultures (e.g. incel forums, manosphere).	Studies measuring cybervictimisation (unless they explicitly included reference to misogyny)<br>Studies measuring exposure to misogyny or sexual harassment in an offline context only.
**Outcome**	Studies measuring mental health outcomes including but not limited to:<br>– Depression<br>– Anxiety<br>– Self-harm thoughts and behaviours<br>– Eating disorder<br>– Self-esteem<br>– Body dissatisfaction<br>– Psychological wellbeing.	Studies that did not measure any mental health outcome.
**Context**	No restrictions were placed on geographic location, language or date.	
**Publication types**	Peer-reviewed studies, pre-prints, conference papers, and theses or dissertations	Letters, commentaries, and editorials which did not include relevant primary empirical data

Appendix 2. Data sought during data extraction

Study characteristics:
- Author
- Title
- Year of publication
- Country)

Population:
- Sample size
- Age
- Sex/gender
- Ethnicity
- Other demographic information
- Follow-up length
- Inclusion criteria
- Subgroup

Methodology:
- Study design
- Study setting
- Aim
- Research question
- Hypothesis

Analytical methods
- Theory/gender conceptualisation
- Exposure
- Exposure measure

- Outcome
- Outcome measure
- Outcome domain
- Covariates)

Results

- Effect size (95% confidence interval and p value/statistical significance)

Discussion

- Conclusions
- Strengths
- Limitations
- Future research suggestions

Appendix 3. Newcastle-Ottawa Quality Assessment Form for Cohort Studies

			**Chao and Yu (1)**	**Copp et al. (2)**	**Daskaluk (3)**	**Dollimore et al. (4)**	**Lawrence et al. (5)**	**Ma (6)**
Selection domain	Representativeness of the exposed cohort	Truly representative		X				
		Somewhat representative	X				X	
		Selected group			X	X		X
		No description of the derivation of the cohort						
	Selection of the non-exposed cohort	Drawn from the same community as the exposed cohort	X	X	X	X	X	X
		Drawn from a different source						
		No description of the derivation of the non-exposed cohort						
	Ascertainment of exposure	Secure record (e.g., surgical record)						
		Structured interview						
		Written self-report	X	X	X	X	X	X
		No description						
		Other						
	Demonstration that outcome of interest was not present at start of study	Yes		X	X	X		X
		No	X				X	
Comparability domain	Comparability of cohorts on the basis of the design or	The study controls for age, sex and marital status		X			X	X

	analysis controlled for confounders	Study controls for other factors (list)			X			
		Cohorts are not comparable on the basis of the design or analysis controlled for confounders				X		
Outcome domain	Assessment of outcome	Independent blind assessment						
		Record linkage						
		Self-report	X	X	X	X	X	X
		No description						
		Other						
	Was follow-up long enough for outcomes to occur?	Yes	X	X	X		X	X
		No				X		
		Indicate the median duration of follow-up and a brief rationale for the assessment:	2 years	2 years	4-months	10-weeks	2 years	1 year
	Adequacy of follow-up of cohorts	Complete follow up- all subject accounted for						
		Subjects lost to follow up unlikely to introduce bias- number lost less than or equal to 20% or description of those lost suggested no different from those followed.					X	
		Follow up rate less than 80% and no description of those lost	X	X	X	X		X
		No statement						
Rating			Fair	Fair	Fair	Poor	Fair	Fair

Appendix 4. Certainty of evidence GRADE assessment

**GRADE domain***	**Judgement**	**Concerns about certainty domains**	**Certainty in the evidence**
Methodological limitations of the studies	None of the included studies justified their sample size by conducting a power calculation.<br><br>Newcastle–Ottawa assessment indicated the six included studies were generally of moderate (fair) methodological quality with the exception of Dollimore et al., (2025) which was rated poor (1).<br><br>All studies used self-reported outcome measures, and follow-up durations varied widely from 10 weeks (1) to two years (2-4), with Dollimore et al. (2025) judged too short for outcomes to be observed<br><br>Loss to follow-up exceeded 20% in five studies without adequate description of attrition (1-3, 5, 6). This contributes to risk of bias and underpinning the overall classification of evidence as predominantly fair but not robust.<br><br>Therefore, we judged the studies included in this review to have borderline serious borderline methodological limitations.	Serious, borderline	Moderate<br>⨁OOO<br>(due to serious borderline risk of bias, and inconsistency)
Indirectness	The studies all provide direct evidence to the research question of this review.<br><br>However, definitions of online misogyny varied considerably; Online sexual harassment victimization (3) Cyber Sexual Aggression (5) cyber sexual harassment (4); sexual harassment on social media (1), and cyber/online pornography (2, 6). Even within these categories, measures were inconsistent, capturing different combinations of behaviours and experiences. Outcomes and analytic approaches were similarly heterogeneous, limiting comparability and precluding quantitative synthesis.	Not serious, borderline	

	Furthermore, two studies did not report sex/gender specific results for girls/young women/females (4, 6).		
Imprecision	The total number of participants involved in each study ranged from 207 (1) to 1,838 (2).<br><br>Samples were large in four studies (n≈1,000-3,000)(2-4, 6) and represented different cultural contexts. Two studies were considerably smaller (207 and 143 at follow-up, respectively)(1, 5).<br><br>One study had sex/gender imbalance and comprised of less than 40% females (2). The overrepresentation of males in the sample introduces a risk of studies detecting an association in the male subgroup, but not in the female subgroup, however this imprecision may be limited by this study's larger sample size of 1,838.	Not serious	
Inconsistency	Evidence of direct effects were mixed, potentially indicating inconsistency.	Serious, borderline	
Publication bias	One of the included studies was an unpublished dissertation, precluding publication bias. Publication bias is not suspected of the included studies due to their mixed findings..	Not suspected	

*Following Murad et al.'s guidance on using GRADE to rate the certainty of evidence when narrative synthesis is conducted instead of meta-analysis (7).

1. Chao C-M, Yu T-K. Internet use and adolescents' physical and mental health: The mediating role of self-consciousness and peer relationships. International Journal of Mental Health and Addiction. 2021:No Pagination Specified.
2. Copp JE, Mumford EA, Taylor BG. Online sexual harassment and cyberbullying in a nationally representative sample of teens: Prevalence, predictors, and consequences. Journal of Adolescence. 2021;93:202-11.

3. Daskaluk S. #metoo: Effects of cyber sexual aggression victimization on women's health and relationship quality. Dissertation Abstracts International: Section B: The Sciences and Engineering. 2022;83(7-B):No Pagination Specified.
4. Dollimore K, Hurst M, Cassarly JA, Bell BT. Cross-sectional and longitudinal relationships between young student women's experiences of everyday sexual harassment on social media and self-objectification, body shame, and personal safety anxiety. Psychology of Popular Media. 2025;14(3):478-83.
5. Lawrence TI, Wojciechowski T, Lee K, Amador B. Longitudinal comorbidity of anxiety and depressive symptoms: The risk of cyber sexual harassment. Child Psychiatry and Human Development. 2025:No Pagination Specified.
6. Ma CM. Relationships between exposure to online pornography, psychological well-being and sexual permissiveness among Hong Kong Chinese adolescents: A three-wave longitudinal study. Applied Research in Quality of Life. 2019;14(2):423-39.
7. Murad MH, Mustafa RA, Schünemann HJ, Sultan S, Santesso N. Rating the certainty in evidence in the absence of a single estimate of effect. Evid Based Med. 2017;22(3):85-7.
