# Supplementary material for: Is exposure to online misogyny associated with mental health outcomes in girls and young women? A systematic review of longitudinal studies
Source: Womens Health (Lond). 2026 Sep 23;22:17455057261485065. doi: 10.1177/17455057261485065 (PMC13735633; doi:10.1177/17455057261485065)
Supplement: Supplementary file 3 [file sj-pdf-3-whe-10.1177_17455057261485065.pdf]

Is exposure to online misogyny associated with mental health outcomes in girls and young women? A systematic review of longitudinal studies

Supplementary file 3: Database searches 4th September 2025

Europe PMC

Search terms

ABSTRACT TITLE:((misogyn* OR "sexual harassment" OR sexi* OR chauvin* OR bigot* OR prejudice OR "revenge porn" OR incel OR "gendered hate" OR trolling OR "intimate image" OR porn OR manosphere) AND (online OR "social media" OR internet OR digital OR gaming OR "video games" OR "online communities") AND ("mental health" OR wellbeing OR "well-being" OR anxiety OR depression OR depressi* OR "psychological distress" OR "self-harm" OR "suicidal ideation" OR "mood disorder*" OR stress) AND (longitudinal OR "longitudinal study" OR "cohort study" OR "panel study" OR "prospective study" OR "follow-up study"))

Results: 69

1. Obradovic T, Murphy D, Fear NT, Sharp ML. Cross-sectional study of the rates of military sexual trauma (MST) and associations with adverse mental health outcomes among UK female ex-service personnel: a study protocol. BMJ Open. 2025 Jun;15(6):e096912. doi: 10.1136/bmjopen-2024-096912. PMID: 40578887; PMCID: PMC12207112.
2. Khadka S, Limbu A, Chalise A, Pandey S, Paudel S. Cyberbullying victimisation and its association with depression, anxiety and stress among female adolescents in Deumai Municipality, Nepal: a cross-sectional survey. BMJ Open. 2024 Oct;14(10):e081797. doi: 10.1136/bmjopen-2023-081797. PMID: 39438093; PMCID: PMC11499772.
3. Prkno D, Grafe N, Schulz MS, Kiess W, Poulain T. Children's and adolescents' negative internet experiences and the association with quality of life and behavioural difficulties: a cross-sectional study. BMJ Paediatr Open. 2025 Apr;9(1):e003135. doi: 10.1136/bmjpo-2024-003135. PMID: 40246585; PMCID: PMC12007062.
4. Bansal S, Garg N, Singh J, Van Der Walt F. Cyberbullying and mental health: past, present and future. Front Psychol. 2023;14:1279234. doi: 10.3389/fpsyg.2023.1279234. PMID: 38288359; PMCID: PMC10823540.
5. FREE PAPERS Indian J Psychiatry. 2025 Jan;67(Suppl 1):S24-S95. PMCID: PMC11932184.
6. Pilgaard F, Östergren PO, Priebe G, Agardh A. Workplace sexual harassment is associated with poor mental well-being among employees at a large Swedish university. Glob Health Action. 2025 Dec;18(1):2465050. doi: 10.1080/16549716.2025.2465050. PMID: 39969584; PMCID: PMC11841154.
7. Matthews T, Arseneault L, Bryan BT, Fisher HL, Gray R, Henchy J, Moffitt TE, Odgers CL. Social media use, online experiences, and loneliness among young adults: A cohort study. Ann N Y Acad Sci. 2025 Jun;1548(1):194-205. doi: 10.1111/nyas.15370. PMID: 40350583; PMCID: PMC12220285.

8. Somé NH, Noormohammadpour P, Lange S. The use of machine learning on administrative and survey data to predict suicidal thoughts and behaviors: a systematic review. Front Psychiatry. 2024;15:1291362. doi: 10.3389/fpsyt.2024.1291362. PMID: 38501090; PMCID: PMC10944962.
9. Wang X, Peng P, Liu Y, Yang WF, Chen S, Wang Y, Yang Q, Li M, Wang Y, Hao Y, He L, Wang Q, Zhang J, Ma Y, He H, Zhou Y, Long J, Qi C, Tang YY, Liao Y, Tang J, Wu Q, Liu T. Workplace violence inflicted by patients or their family members/visitors and its relationship with suicidal ideation among undergraduate medical students during clinical training in China. Ann Med. 2023;55(2):2295027. doi: 10.1080/07853890.2023.2295027. PMID: 38146746; PMCID: PMC10763877.
10. Nielsen MBD, Skov SS, Grundtvig G, Folker AP, Rugulies R, Tybjerg Aldrich P, Clausen T, Madsen IEH. Workplace sexual harassment: a qualitative study of the self-labelling process among employees in Denmark. Int J Qual Stud Health Well-being. 2024 Dec;19(1):2324990. doi: 10.1080/17482631.2024.2324990. PMID: 38648665; PMCID: PMC11036896.
11. Amadori A, Real AG, Brighi A, Russell ST. An Intersectional Perspective on Cyberbullying: Victimization Experiences Among Marginalized Youth. J Adolesc. 2025 Jun;97(4):931-940. doi: 10.1002/jad.12466. PMID: 39827373; PMCID: PMC12128902.
12. Nelson S, Ayaz B, Baumann AL, Dozois G. A gender-based review of workplace violence amongst the global health workforce-A scoping review of the literature. PLOS Glob Public Health. 2024;4(7):e0003336. doi: 10.1371/journal.pgph.0003336. PMID: 38954697; PMCID: PMC11218983.
13. Liu F, Chong ESK. Effects of sexual orientation-based prejudice and discrimination in family of origin on depressive symptoms and life satisfaction in a Chinese sample: Reciprocal and authoritarian filial piety as moderators. Fam Process. 2024 Dec;63(4):2416-2433. doi: 10.1111/famp.13012. PMID: 38784985; PMCID: PMC11659091.
14. Al-Mamun F, Kaggwa MM, Hosen I, Sikder MT, Griffiths MD, Mamun MA. Suicide related to the COVID-19 pandemic in India: A systematic review. Discov Psychol. 2023;3(1):2. doi: 10.1007/s44202-022-00063-1. PMID: 40477694; PMCID: PMC9831013.
15. Thorell LB, Burén J, Ström Wiman J, Sandberg D, Nutley SB. Longitudinal associations between digital media use and ADHD symptoms in children and adolescents: a systematic literature review. Eur Child Adolesc Psychiatry. 2024 Aug;33(8):2503-2526. doi: 10.1007/s00787-022-02130-3. PMID: 36562860; PMCID: PMC11272698.
16. Novitzky P, Janssen J, Kokkeler B. A systematic review of ethical challenges and opportunities of addressing domestic violence with AI-technologies and online tools. Heliyon. 2023 Jun;9(6):e17140. doi: 10.1016/j.heliyon.2023.e17140. PMID: 37342580; PMCID: PMC10277589.
17. Rogers MM, Fisher C, Ali P, Allmark P, Fontes L. Technology-Facilitated Abuse in Intimate Relationships: A Scoping Review. Trauma Violence Abuse. 2023 Oct;24(4):2210-2226. doi: 10.1177/15248380221090218. PMID: 35537445; PMCID: PMC10486147.
18. Knowles G, Stanyon D, Yamasaki S, Miyashita M, Gayer-Anderson C, Endo K, Usami S, Niimura J, Nakajima N, Baba K, TTC Young Persons Advisory Group, Richards T, Kitisu J, Hashi A, Clement-Gbede KS, Tettey N, Davis S, Lowis K, Buckley V, Moreno-Agostino D, Putzgruber E, Crudgington H, Woodhead C, Sawyer K, Keyes KM, Dyer J, Ando S,

Kasai K, Hiraiwa-Hasegawa M, Morgan C, Nishida A. Gender inequalities in trajectories of depressive symptoms among young people in London and Tokyo: a longitudinal cross-cohort study. medRxiv; 2023. doi: 10.1101/2023.11.22.23298823. PPR:PPR764270.

19. Ho L, Cheung A, Chan C, Wong E, Tam W, Chien W. Empowering workplace allies for lesbian, gay, bisexual, and transgender employees to prevent and minimize psychological distress: A scoping review Cochrane Evid Synth Methods. 2023 Jun;1(4):. PMCID: PMC11795962.
20. Ranganathan M, Wamoyi J, Pearson I, Stöckl H. Measurement and prevalence of sexual harassment in low- and middle-income countries: a systematic review and meta-analysis. BMJ Open. 2021 Jun;11(6):e047473. doi: 10.1136/bmjopen-2020-047473. PMID: 34168030; PMCID: PMC8231049.
21. Kwan I, Dickson K, Richardson M, MacDowall W, Burchett H, Stansfield C, Brunton G, Sutcliffe K, Thomas J. Cyberbullying and Children and Young People's Mental Health: A Systematic Map of Systematic Reviews. Cyberpsychol Behav Soc Netw. 2020 Feb;23(2):72-82. doi: 10.1089/cyber.2019.0370. PMID: 31977251; PMCID: PMC7044782.
22. Posters Eur J Psychotraumatol. 2023 Jun;14(Suppl 1):. PMCID: PMC10269383.
23. Sivertsen B, Knudsen AKS, Kirkøen B, Skogen JC, Lagerstrøm BO, Lønning KJ, Kessler RC, Reneflot A. Prevalence of mental disorders among Norwegian college and university students: a population-based cross-sectional analysis. Lancet Reg Health Eur. 2023 Nov;34:100732. doi: 10.1016/j.lanepe.2023.100732. PMID: 37927428; PMCID: PMC10624983.
24. Barakat S, McLean SA, Bryant E, Le A, Marks P, National Eating Disorder Research Consortium, Touyz S, Maguire S. Risk factors for eating disorders: findings from a rapid review. J Eat Disord. 2023 Jan;11(1):8. doi: 10.1186/s40337-022-00717-4. PMID: 36650572; PMCID: PMC9847054.
25. Fulantelli G, Taibi D, Scifo L, Schwarze V, Eimler SC. Cyberbullying and Cyberhate as Two Interlinked Instances of Cyber-Aggression in Adolescence: A Systematic Review. Front Psychol. 2022;13:909299. doi: 10.3389/fpsyg.2022.909299. PMID: 35712182; PMCID: PMC9196243.
26. Xuereb S. Emotions, perceived threat, prejudice, and attitudes towards helping Ukrainian, Syrian, and Somali asylum seekers. PLoS One. 2023;18(9):e0290335. doi: 10.1371/journal.pone.0290335. PMID: 37703255; PMCID: PMC10499223.
27. Ning M, Chen Z, Yang J, Li X, Yu Q, Huang C, Li Y, Tian Y. Development and validation of a nomogram for predicting high-burnout risk in nurses. J Clin Nurs. 2025 Apr;34(4):1338-1350. doi: 10.1111/jocn.17210. PMID: 38736145; PMCID: PMC11933524.
28. Konkolÿ Thege B, Robitaille C, Mahmoud L, Kinzel E, Qamar R, Hartmann-Boyce J, Choy O. The Efficacy of Nutritional Interventions in Reducing Childhood/Youth Aggressive and Antisocial Behavior: A Mixed-Methods Systematic Review and Meta-Analysis Campbell Syst Rev. 2025 Aug;21(3):. PMCID: PMC12335748.
29. Pakpahan C, Darmadi D, Agustinus A, Rezano A. Framing and understanding the whole aspect of oral sex from social and health perspectives: a narrative review. F1000Res. 2022;11:177. doi: 10.12688/f1000research.108675.3. PMID: 36035886; PMCID: PMC9377381.

30. Prictor M. Saving trouble, saving time: the role and impact of healthcare consultation recordings in Australian legal proceedings. Med Law Rev. 2025 Jan;33(1):fwaf012. doi: 10.1093/medlaw/fwaf012. PMID: 40085962; PMCID: PMC11908765.
31. 2024 International Congress on Integrative Medicine & Health Abstracts Glob Adv Integr Med Health. 2024 Jan;13:. PMCID: PMC11047233.
32. BOOK OF POSTERS Can J Pain. 2024 Oct;8(3):. PMCID: PMC11540093.
33. Kourti A, Stavridou A, Panagouli E, Psaltopoulou T, Spiliopoulou C, Tsolia M, Sergentanis TN, Tsitsika A. Domestic Violence During the COVID-19 Pandemic: A Systematic Review. Trauma Violence Abuse. 2023 Apr;24(2):719-745. doi: 10.1177/15248380211038690. PMID: 34402325; PMCID: PMC10011925.
34. Argyriou A, Goldsmith KA, Rimes KA. Mediators of the Disparities in Depression Between Sexual Minority and Heterosexual Individuals: A Systematic Review. Arch Sex Behav. 2021 Apr;50(3):925-959. doi: 10.1007/s10508-020-01862-0. PMID: 33689086; PMCID: PMC8035121.
35. ESICM LIVES 2024. Barcelona, Spain. 5–9 October 2024. Intensive Care Med Exp. 2024 Oct;12(Suppl 1):87. doi: 10.1186/s40635-024-00658-z. PMID: 39361093; PMCID: PMC11450122.
36. O'Doherty L, Whelan M, Carter GJ, Brown K, Tarzia L, Hegarty K, Feder G, Brown SJ. Psychosocial interventions for survivors of rape and sexual assault experienced during adulthood. Cochrane Database Syst Rev. 2023 Oct;10:CD013456. doi: 10.1002/14651858.cd013456.pub2. PMID: 37795783; PMCID: PMC10552071.
37. Schacter DL. Memory Sins in Applied Settings: What Kind of Progress? J Appl Res Mem Cogn. 2022 Dec;11(4):445-460. doi: 10.1037/mac0000078. PMID: 37035272; PMCID: PMC10077946.
38. NNVAWI Abstracts. Glob Qual Nurs Res. 2020 Jan-Dec;7:2333393620932494. doi: 10.1177/2333393620932494. Erratum in: Glob Qual Nurs Res. 2020 Oct 8;7:2333393620967636. doi: 10.1177/2333393620967636. PMID: 32656298; PMCID: PMC7331758.
39. Karsay K, Knoll J, Matthes J. Sexualizing Media Use and Self-Objectification: A Meta-Analysis. Psychol Women Q. 2018 Mar;42(1):9-28. doi: 10.1177/0361684317743019. PMID: 29527090; PMCID: PMC5833025.
40. Kapetanovic S, Gurdal S, Ander B, Sorbring E. Reported Change in Adolescent Psychosocial Functioning during the Covid-19 Outbreak. Research Square; 2020. doi: 10.21203/rs.3.rs-99461/v1. PPR:PPR232474.
41. ABSTRACTS FOR ANCIPS 2020 Indian J Psychiatry. 2020 Jan;62(Suppl 1):S74-S122. PMCID: PMC6991111.
42. Coleman E, Radix AE, Bouman WP, Brown GR, de Vries ALC, Deutsch MB, Ettner R, Fraser L, Goodman M, Green J, Hancock AB, Johnson TW, Karasic DH, Knudson GA, Leibowitz SF, Meyer-Bahlburg HFL, Monstrey SJ, Motmans J, Nahata L, Nieder TO, Reisner SL, Richards C, Schechter LS, Tangpricha V, Tishelman AC, Van Trotsenburg MAA, Winter S, Ducheny K, Adams NJ, Adrián TM, Allen LR, Azul D, Bagga H, Başar K, Bathory DS, Belinky JJ, Berg DR, Berli JU, Bluebond-Langner RO, Bouman MB, Bowers ML, Brassard PJ, Byrne J, Capitán L, Cargill CJ, Carswell JM, Chang SC, Chelvakumar G, Corneil T, Dalke KB, De Cuypere G, de Vries E, Den Heijer M, Devor AH, Dhejne C, D'Marco A, Edmiston EK, Edwards-Leeper L, Ehrbar R, Ehrensaft D, Eisfeld J, Elaut E, Erickson-Schroth L, Feldman JL, Fisher AD, Garcia MM, Gijs L, Green SE, Hall BP, Hardy TLD, Irwig MS, Jacobs LA, Janssen AC, Johnson K, Klink DT, Kreukels BPC, Kuper LE,

Kvach EJ, Malouf MA, Massey R, Mazur T, McLachlan C, Morrison SD, Mosser SW, Neira PM, Nygren U, Oates JM, Obedin-Maliver J, Pagkalos G, Patton J, Phanuphak N, Rachlin K, Reed T, Rider GN, Ristori J, Robbins-Cherry S, Roberts SA, Rodriguez-Wallberg KA, Rosenthal SM, Sabir K, Safer JD, Scheim AI, Seal LJ, Sehoole TJ, Spencer K, St Amand C, Steensma TD, Strang JF, Taylor GB, Tilleman K, T'Sjoen GG, Vala LN, Van Mello NM, Veale JF, Vencill JA, Vincent B, Wesp LM, West MA, Arcelus J. Standards of Care for the Health of Transgender and Gender Diverse People, Version 8. Int J Transgend Health. 2022;23(Suppl 1):S1-S259. doi: 10.1080/26895269.2022.2100644. PMID: 36238954; PMCID: PMC9553112.
43. Abstract Supplement Abstracts from AIDS 2022, the 24th International AIDS Conference, 29 July - 2 August 2022, Montreal, Canada & Virtual. J Int AIDS Soc. 2022 Aug;25 Suppl 3:e25935. doi: 10.1002/jia2.25935. PMID: 35916394; PMCID: PMC9344833.
44. ABSTRACTS FOR ANCIPS 2020 Indian J Psychiatry. 2020 Jan;62(Suppl 1):S30-S73. PMCID: PMC6991107.
45. 25th Congress of the World Association for Sexual Health (WAS) and SASHA (South African Sexual Health Association) Int J Sex Health. 2022 Apr;34(Suppl 1):1-331. PMCID: PMC10903548.
46. Ross D, Akil M, Williams D, Szalay D, Wooster D, Wooster E, Malin G, Babenko O, Matlow A, Lee S, Maniate J, Harvey K, Do V, Kahlke R, Monteiro S, Chan T, Burrell A, Kirpalani A, Peebles E, McMurren C, Giroux R, Dumont M, Osmack M, Ellaway R, Wyatt T, La T, Nimmon L, Young M, Cristancho S, Dubé T, Leblanc C, Moloo H, Maniate J, Chan M, Dickinson B, Bracken K, Towle A, Descoteaux A, Lauscher D, Jackson M, Gibson K, Zucker M, Bang P, Goldstein C, Lewis M, Do V, Menezes A, Salami E, Maniate J, Bains S, Lam J, Chen R, Farrugia P, Esmail A, James L, O'Campo P, Osei-Twum J, Olarewaju G, Razack S, Jafarian F, Szent-Györgyi A, Benoit S, Aristide D. International Congress on Academic Medicine: 2024 medical education abstracts Le congrès international de médecine universitaire: résumes d'éducation médicale 2024 Can Med Educ J. 2024 Jun;15(3):132-364. PMCID: PMC11302768.
47. Virgara R, Phillips A, Lewis LK, Baldock K, Wolfenden L, Ferguson T, Richardson M, Okely A, Beets M, Maher C. Interventions in outside-school hours childcare settings for promoting physical activity amongst schoolchildren aged 4 to 12 years. Cochrane Database Syst Rev. 2021 Sep;9:CD013380. doi: 10.1002/14651858.cd013380.pub2. PMID: 34694005; PMCID: PMC8543676.
48. Abstract Supplement Oral Abstracts from the 23rd International AIDS Conference, 6-10 July 2020. J Int AIDS Soc. 2020 Jul;23 Suppl 4:e25547. doi: 10.1002/jia2.25547. PMID: 32649039; PMCID: PMC7350918.
49. Canadian Conference on Medical Education 2022 abstracts. Can Med Educ J. 2022 May;13(2):105-251. doi: 10.36834/cmej.75002. PMID: 35572020; PMCID: PMC9099163.
50. Abstracts from the 2024 Annual Meeting of the Society of General Internal Medicine J Gen Intern Med. 2024 Jun;39(Suppl 2):137-1008. PMCID: PMC11231130.
51. Meyer SR, Stöckl H, Vorfeld C, Kamenov K, García-Moreno C. A scoping review of measurement of violence against women and disability. PLoS One. 2022;17(1):e0263020. doi: 10.1371/journal.pone.0263020. PMID: 35100320; PMCID: PMC8803172.
52. Supplement J Athl Train. 2020 Jun;55(6 Suppl):S-1-S-378. PMCID: PMC7745344.

53. Program Abstracts from the GSA 2018 Annual Scientific Meeting "The Purposes of Longer Lives". Innov Aging. 2018 Nov;2(Suppl 1):1-877. doi: 10.1093/geroni/igy023. PMID: 30480150; PMCID: PMC6232832.
54. Huang PH. Put More Women in Charge and Other Leadership Lessons from COVID-19. SSRN; 2020. doi: 10.2139/ssrn.3604783. PPR:PPR244573.
55. SYMPOSIUM Indian J Psychiatry. 2019 Jan;61(Suppl 3):S390-S439. PMCID: PMC6366286.
56. Atti 55° Congresso Nazionale SItI Padova, 28 settembre - 1 ottobre, 2022. J Prev Med Hyg. 2022;63(2 Suppl 1):E1-E443. doi: 10.15167/2421-4248/jpmh2022.63.2s1. PMID: 36338896; PMCID: PMC9615638.
57. Proceedings of the 15th Annual Conference on the Science of Dissemination and Implementation in Health. Implement Sci. 2023 Oct;18(Suppl 3):48. doi: 10.1186/s13012-023-01299-8. PMID: 37794475; PMCID: PMC10552189.
58. Oral abstracts of the 22nd International AIDS Conference, 23-27 July 2018, Amsterdam, the Netherlands. J Int AIDS Soc. 2018 Jul;21 Suppl 6:e25148. doi: 10.1002/jia2.25148. PMID: 30051631; PMCID: PMC6062436.
59. Abstracts from the 2022 Annual Meeting of the Society of General Internal Medicine J Gen Intern Med. 2022 Jun;37(Suppl 2):129-664. PMCID: PMC9205280.
60. Abstracts of the 52nd Annual Conference of the Italian Society of Neurology. Neurol Sci. 2022 Dec:1-530. doi: 10.1007/s10072-022-06531-9. PMID: 36454442; PMCID: PMC9713129.
61. Abstracts from the 2021 Annual Meeting of the Society of General Internal Medicine. J Gen Intern Med. 2021 Jul;36(Suppl 1):1-469. doi: 10.1007/s11606-021-06830-5. PMID: 34297318; PMCID: PMC8299734.
62. Abstracts from the 2020 Annual Meeting of the Society of General Internal Medicine. J Gen Intern Med. 2020 Jul;35(Suppl 1):1-779. doi: 10.1007/s11606-020-05890-3. PMID: 32725471; PMCID: PMC7385936.
63. Abstracts from the 2019 Annual Meeting of the Society of General Internal Medicine. J Gen Intern Med. 2019 May;34(Suppl 2):99-867. doi: 10.1007/s11606-019-05007-5. PMID: 31090026; PMCID: PMC6582171.
64. Full Issue PDF JACC Case Rep. 2020 Oct;2(12):I-CCXXI. PMCID: PMC7721314.
65. Abstracts Int J Legal Med. 2021 Jan;135(Suppl 1):11-119. PMCID: PMC8123097.
66. Abstracts from the 2016 Society of General Internal Medicine Annual Meeting. J Gen Intern Med. 2016 May;31 Suppl 2:85-922. doi: 10.1007/s11606-016-3657-7. PMID: 27114361; PMCID: PMC4870402.
67. Abstracts from the 35th Annual Meeting of the Society of General Internal Medicine. May 9-12, 2012. Orlando, Florida, USA. J Gen Intern Med. 2012 Jul;27 Suppl 2:S99-574. doi: 10.1007/s11606-012-2038-0. PMID: 22528616; PMCID: PMC6730975.
68. Abstracts of the Society of General Internal Medicine 32nd Annual Meeting. Miami Beach, Florida, USA. May 13 -16, 2009. J Gen Intern Med. 2009 Apr;24 Suppl 1:S1-380. doi: 10.1007/s11606-009-0963-3. PMID: 19350330; PMCID: PMC2670986.
69. 111th Annual Meeting of the American Association of Colleges of Pharmacy, Seattle, Washington, July 10-14, 2010 Am J Pharm Educ. 2010 Jun;74(5):. PMCID: PMC2907861.

PubMed

Search terms

(misogyn*[Title/Abstract] OR "sexual harassment"[MeSH Terms] OR "sexual harassment"[Title/Abstract] OR sexism[Title/Abstract] OR chauvin*[Title/Abstract] OR bigot*[Title/Abstract] OR prejudice[MeSH Terms] OR prejudice[Title/Abstract] OR "revenge porn"[Title/Abstract] OR incel[Title/Abstract] OR "gendered hate"[Title/Abstract] OR trolling[Title/Abstract] OR "intimate image"[Title/Abstract] OR porn[Title/Abstract] OR manosphere[Title/Abstract]) AND ("internet"[MeSH Terms] OR "social media"[MeSH Terms] OR "video games"[MeSH Terms] OR online[Title/Abstract] OR digital[Title/Abstract] OR gaming[Title/Abstract] OR "social media"[Title/Abstract]) AND ("mental health"[MeSH Terms] OR "mental health"[Title/Abstract] OR "well-being"[Title/Abstract] OR wellbeing[Title/Abstract] OR anxiety[MeSH Terms] OR anxiety[Title/Abstract] OR depression[MeSH Terms] OR depressi*[Title/Abstract] OR "psychological distress"[Title/Abstract] OR "self-harm"[MeSH Terms] OR "self-harm"[Title/Abstract] OR "self injurious behavior"[Title/Abstract] OR "suicidal ideation"[MeSH Terms] OR "suicidal ideation"[Title/Abstract] OR "mood disorders"[MeSH Terms] OR "mood disorder*"[Title/Abstract] OR stress[MeSH Terms] OR stress[Title/Abstract]) AND ("longitudinal studies"[MeSH Terms] OR "cohort studies"[MeSH Terms] OR "follow-up studies"[MeSH Terms] OR longitudinal[Title/Abstract] OR cohort[Title/Abstract] OR "panel study"[Title/Abstract] OR "prospective study"[Title/Abstract])

Results: 38

1: Del Toro J, Wang MT. Online Racism and Mental Health Among Black American Adolescents in 2020. J Am Acad Child Adolesc Psychiatry. 2023 Jan;62(1):25-36.e8. doi: 10.1016/j.jaac.2022.07.004. Epub 2022 Jul 19. PMID: 35868431; PMCID: PMC9805489.

2: Brookman R, Bird F, Harris CB, Grant KA. Social Anxiety Disorder: Associations with Peer-Liking, Discrimination, and Prejudicial Feelings in Early Adolescent Girls. Child Psychiatry Hum Dev. 2023 Oct;54(5):1231-1241. doi: 10.1007/s10578-022-01336-5. Epub 2022 Feb 23. PMID: 35195808; PMCID: PMC8863903.

3: Egorov AY. Povedencheskie (nekhimicheskie) zavisimosti i COVID-19 [Behavioral (non-chemical) addictions and COVID-19]. Zh Nevrol Psikhiatr Im S S Korsakova. 2023;123(1):41-46. Russian. doi: 10.17116/jnevro202312301141. PMID: 36719118.

4: Power S, Biondi A, Saatci I, Bennett K, Mahadevan J, Januel AC, Singhara Na Ayudhaya SP, Agid R. Women in neurointervention, a gender gap? Results of a prospective online survey. Interv Neuroradiol. 2022 Jun;28(3):311-322. doi: 10.1177/15910199211030783. Epub 2021 Sep 13. PMID: 34516279; PMCID: PMC9185108.

5: Huang CY, Tsai W. Asian American parents' experiences of stress, discrimination, and mental health during COVID-19. Fam Syst Health. 2023

Mar;41(1):68-77. doi: 10.1037/fsh0000715. Epub 2022 May 19. PMID: 35588384.

6: Copp JE, Mumford EA, Taylor BG. Online sexual harassment and cyberbullying in a nationally representative sample of teens: Prevalence, predictors, and consequences. J Adolesc. 2021 Dec;93:202-211. doi: 10.1016/j.adolescence.2021.10.003. Epub 2021 Nov 19. PMID: 34801812.

7: Martin R, Williams C, O'Neill D. Retrospective analysis of attitudes to ageing in the Economist: apocalyptic demography for opinion formers. BMJ. 2009 Dec 8;339:b4914. doi: 10.1136/bmj.b4914. PMID: 19995819; PMCID: PMC2790575.

8: Barbier JM, Carrard V, Schwarz J, Berney S, Clair C, Berney A. Exposure of medical students to sexism and sexual harassment and their association with mental health: a cross-sectional study at a Swiss medical school. BMJ Open. 2023 Apr 27;13(4):e069001. doi: 10.1136/bmjopen-2022-069001. PMID: 37105707; PMCID: PMC10151891.

9: Parke R, Bates S, Carey M, Cavadino A, Ferguson A, Hammond N, Joyce F, Kirby S, Moeke-Maxwell T, Nona F, Mason K. Bullying, discrimination, and sexual harassment among intensive care unit nurses in Australia and New Zealand: An online survey. Aust Crit Care. 2023 Jan;36(1):10-18. doi: 10.1016/j.aucc.2022.08.010. Epub 2022 Oct 6. PMID: 36210282.

10: Njoroge WFM, White LK, Waller R, Forkpa M, Himes MM, Morgan K, Seidlitz J, Chaiyachati BH, Barzilay R, Kornfield SL, Parish-Morris J, Rodriguez Y, Riis V, Burris HH, Elovitz MA, Gur RE. Association of COVID-19 and Endemic Systemic Racism With Postpartum Anxiety and Depression Among Black Birthing Individuals. JAMA Psychiatry. 2022 Jun 1;79(6):600-609. doi: 10.1001/jamapsychiatry.2022.0597. PMID: 35416928; PMCID: PMC9008565.

11: Lawrence TI, Wojciechowski T, Lee K, Amador B. Longitudinal Comorbidity of Anxiety and Depressive Symptoms: The Risk of Cyber Sexual Harassment. Child Psychiatry Hum Dev. 2025 Mar 5. doi: 10.1007/s10578-025-01820-8. Epub ahead of print. PMID: 40042776.

12: Peng K, Zhu X, Gillespie A, Wang Y, Gao Y, Xin Y, Qi J, Ou J, Zhong S, Zhao L, Liu J, Wang C, Chen R. Self-reported Rates of Abuse, Neglect, and Bullying Experienced by Transgender and Gender-Nonbinary Adolescents in China. JAMA Netw Open. 2019 Sep 4;2(9):e1911058. doi: 10.1001/jamanetworkopen.2019.11058. PMID: 31490542; PMCID: PMC6735403.

13: Jonsdottir SD, Hauksdottir A, Aspelund T, Jakobsdottir J, Runarsdottir H, Gudmundsdottir B, Tomasson G, Valdimarsdottir UA, Halldorsdottir T, Thordardottir EB. Risk factors for workplace sexual harassment and violence among a national cohort of women in Iceland: a cross-sectional study. Lancet Public Health. 2022 Sep;7(9):e763-e774. doi: 10.1016/S2468-2667(22)00201-8. PMID: 36057275; PMCID: PMC9449977.

14: Evans-Lacko S, London J, Japhet S, Rüsch N, Flach C, Corker E, Henderson C, Thornicroft G. Mass social contact interventions and their effect on mental health related stigma and intended discrimination. BMC Public Health. 2012 Jun 28;12:489. doi: 10.1186/1471-2458-12-489. PMID: 22742085; PMCID: PMC3461459.

15: Jonsdottir SD, Thordardottir EB, Valdimarsdottir UA, Halldorsdottir T, Gudnadottir SA, Jakobsdottir J, Runarsdottir H, Tomasson G, Aspelund T, Hauksdottir A. Sexual violence in the workplace and associated health outcomes: a nationwide, cross-sectional analysis of women in Iceland. Lancet Public Health. 2024 Jun;9(6):e365-e375. doi: 10.1016/S2468-2667(24)00075-6. PMID: 38821683; PMCID: PMC11163433.

16: Israel T, Goodman JA, Merrill CRS, Lin YJ, Kary KG, Matsuno E, Choi AY. Reducing Internalized Homonegativity: Refinement and Replication of an Online Intervention for Gay Men. J Homosex. 2021 Dec 6;68(14):2393-2409. doi: 10.1080/00918369.2020.1804262. Epub 2020 Oct 1. PMID: 33001000.

17: de Macêdo PFC, Brito E, de Magalhães Cunha C, da Purificação Nazaré Araújo M, Martins PC, de Santana MLP. Weight stigma and disordered eating behaviors during the COVID-19 pandemic: the mediating role of weight gain concern and psychological distress. Eat Weight Disord. 2023 Sep 27;28(1):78. doi: 10.1007/s40519-023-01608-6. PMID: 37759035; PMCID: PMC10533574.

18: Hayes S, Lonergan A, Trompeter N, Bussey K, Hay P, Mitchison D. Longitudinal relationship between experience of sexual harassment and 2-year body image and weight outcomes in adolescence: mediation through self-objectification and psychological distress. Eat Weight Disord. 2022 Dec;27(8):3095-3108. doi: 10.1007/s40519-022-01432-4. Epub 2022 Jul 14. PMID: 35834106; PMCID: PMC9803756.

19: Fischer VJ, Bravo RG, Brunnet AE, Michielsen K, Tucker JD, Campbell L, Vögele C. Sexual satisfaction and sexual behaviors during the COVID-19 pandemic: results from the International Sexual Health And REproductive (I-SHARE) health survey in Luxembourg. BMC Public Health. 2022 Jun 3;22(1):1108. doi: 10.1186/s12889-022-13509-x. PMID: 35658847; PMCID: PMC9163902.

20: Printz Pereira D, Dominguez Perez S, Milan S. U.S. mothers' appraisals of the race-related events of 2020: Implications for the course of maternal posttraumatic stress disorder symptoms. J Trauma Stress. 2023 Apr;36(2):272-284. doi: 10.1002/jts.22904. Epub 2023 Jan 2. PMID: 36593587.

21: Carels RA, Miller JC, Hlavka R, Shonrock AMT. The interaction between BMI, weight criticism, weight bias, and psychological and relational outcomes within marriage. Eat Weight Disord. 2022 Jun;27(5):1887-1893. doi: 10.1007/s40519-021-01324-z. Epub 2021 Oct 26. PMID: 34704191.

22: Bergman YS, Segel-Karpas D. Aging anxiety, loneliness, and depressive

symptoms among middle-aged adults: The moderating role of ageism. J Affect
Disord. 2021 Jul 1;290:89-92. doi: 10.1016/j.jad.2021.04.077. Epub 2021 May 4.
PMID: 33993085.

23: Buchanan CM, McDougall P. Predicting Psychosocial Maladjustment in Emerging
Adulthood From High School Experiences of Peer Victimization. J Interpers
Violence. 2021 Feb;36(3-4):NP1810-1832NP. doi: 10.1177/0886260518756115. Epub
2018 Jan 31. PMID: 29385870.

24: Lunyera J, Davenport CA, Bhavsar NA, Sims M, Scialla J, Pendergast J, Hall
R, Tyson CC, Russell JSC, Wang W, Correa A, Boulware LE, Diamantidis CJ.
Nondepressive Psychosocial Factors and CKD Outcomes in Black Americans. Clin J
Am Soc Nephrol. 2018 Feb 7;13(2):213-222. doi: 10.2215/CJN.06430617. Epub 2018
Jan 3. PMID: 29298761; PMCID: PMC5967427.

25: Sølvberg N, Torstveit MK, Solstad BE, Mountjoy M, Rosenvinge JH, Pettersen
G, Sundgot-Borgen J. Risk factors for sexual harassment and abuse victimization
among adolescent athletes and non-athletes: A one-year follow-up study. Child
Abuse Negl. 2024 Jan;147:106592. doi: 10.1016/j.chiabu.2023.106592. Epub 2023
Dec 18. PMID: 38113571.

26: Yang SJ, Stewart R, Lee JY, Kim JM, Kim SW, Shin IS, Yoon JS. Prevalence and
correlates of problematic internet experiences and computer-using time: a two-
year longitudinal study in korean school children. Psychiatry Investig. 2014
Jan;11(1):24-31. doi: 10.4306/pi.2014.11.1.24. Epub 2014 Jan 21. PMID: 24605120;
PMCID: PMC3942548.

27: Clary LK, Mendelson T, Webb L, Jackson D, Guerrero Vazquez M, Holifield J,
Musci R, Johnson RM, Sanchez D, Jans M, Watts R. Longitudinal study of
adolescent stress, critical consciousness and resilience trajectories in the
context of structural racism: the RISE Baltimore study protocol. BMJ Open. 2025
May 30;15(5):e094618. doi: 10.1136/bmjopen-2024-094618. PMID: 40447432; PMCID:
PMC12128476.

28: Pijlman V, Boertien E. A Comparative Study of the Help-Seeking Behavior of
Victims of Contact Sexual Violence and Image-Based Sexual Harassment and Abuse.
Violence Against Women. 2024 Sep 27:10778012241283496. doi:
10.1177/10778012241283496. Epub ahead of print. PMID: 39327999.

29: Ruiz RA, Lehavot K, Heffner JL, Kava CM, Ornelas IJ. Associations of Sexual
Orientation-Related Minority and Military Stressors With Past-Year Cigarette Use
Among Lesbian, Gay, and Bisexual Veterans. Nicotine Tob Res. 2024 Jun
21;26(7):852-860. doi: 10.1093/ntr/ntad210. PMID: 37878537; PMCID: PMC11190049.

30: Barnhart WR, Xiao Y, Li Y, Gaggiano C, Jiang Z, Wu S, Cao H, He J. Beyond
Age, BMI, Gender Identity, and Gender Minority Stress, Weight Bias
Internalization Is Uniquely Associated With More Eating and Body Image

Disturbances and Poor Physical and Mental Health in Chinese Gender-Diverse Adults. Int J Eat Disord. 2024 Nov;57(11):2246-2259. doi: 10.1002/eat.24278. Epub 2024 Aug 23. PMID: 39177303.

31: Lin YC, Lin CY, Saffari M, Tsai MC, Chang YH, Strong C, Chen JK, Hsieh YP, Yang YN, Latner JD. Weight stigma is associated with body mass index among college students in Taiwan: the mediated role of internalized weight stigma. BMC Psychol. 2023 Nov 1;11(1):365. doi: 10.1186/s40359-023-01414-w. PMID: 37915074; PMCID: PMC10621093.

32: Sangalli L, Shaddox LM, De Souza GM. Gender Differences in Barriers in Career Development Among a Cohort of Dental Researchers. A Cross-Sectional Study. J Dent Educ. 2025 Aug;89(8):1267-1283. doi: 10.1002/jdd.13832. Epub 2025 Jan 23. PMID: 39853668.

33: Obradovic T, Murphy D, Fear NT, Sharp ML. Cross-sectional study of the rates of military sexual trauma (MST) and associations with adverse mental health outcomes among UK female ex-service personnel: a study protocol. BMJ Open. 2025 Jun 26;15(6):e096912. doi: 10.1136/bmjopen-2024-096912. PMID: 40578887; PMCID: PMC12207112.

34: Nakanishi A, Ichikawa M, Sano Y. Public Discourse Toward Older Drivers in Japan Using Social Media Data From 2010 to 2022: Longitudinal Analysis. JMIR Infodemiology. 2025 Jun 16;5:e69321. doi: 10.2196/69321. PMID: 40523233; PMCID: PMC12209477.

35: Martens L, Virgin N, Hoffarth P, Goodwill J, Derenne N, Eck RV, Klug M, Manocha GD, Jurivich D. Recapitulation of Ageism in Artificial Intelligence-Generated Images: Longitudinal Comparative Study. J Med Internet Res. 2025 Aug 13;27:e68428. doi: 10.2196/68428. PMID: 40803004; PMCID: PMC12349884.

36: Grekin ER. Perceived racism and alcohol consequences among African American and Caucasian college students. Psychol Addict Behav. 2012 Dec;26(4):924-30. doi: 10.1037/a0029593. Epub 2012 Oct 8. PMID: 23046273.

37: McGinley M, Rospenda KM, Liu L, Richman JA. It isn't all just fun and games: Collegiate participation in extracurricular activities and risk for generalized and sexual harassment, psychological distress, and alcohol use. J Adolesc. 2016 Dec;53:152-163. doi: 10.1016/j.adolescence.2016.10.001. Epub 2016 Oct 27. PMID: 27771523; PMCID: PMC5123845.

38: Averett P, Yoon I, Jenkins CL. Older lesbians: experiences of aging, discrimination and resilience. J Women Aging. 2011;23(3):216-32. doi: 10.1080/08952841.2011.587742. PMID: 21767086.

PsycINFO

Search terms

Database(s): **APA PsycInfo** 1806 to August 2025 Week 4
Search Strategy:

#	Searches	Results
1	Misogyny.mp.	804
2	exp Sexual Harassment/	3511
3	exp Sexism/	3802
4	exp Sex Discrimination/	3473
5	exp sex role attitudes/	14425
6	exp Prejudice/	10351
7	exp pornography/	3238
8	misogyn*.ab,ti.	1058
9	"sexual harassment".ab,ti.	4230
10	sexi*.ti,ab.	6620
11	chauvin*.ti,ab.	271
12	bigot*.ti,ab.	424
13	prejudice.ti,ab.	14778
14	"revenge porn".ti,ab.	54
15	"gendered hate".ti,ab.	3
16	trolling.ti,ab.	157
17	"intimate image".ti,ab.	19
18	porn*.ti,ab.	4432
19	manosphere.ti,ab.	40
20	incel*.ti,ab.	1036
21	1 or 2 or 3 or 4 or 5 or 6 or 7 or 8 or 9 or 10 or 11 or 12 or 13 or 14 or 15 or 16 or 17 or 18 or 19 or 20	49078
22	exp online behavior/	1187
23	online.ti,ab.	156144
24	exp social media/	30001
25	"social media".ti,ab.	28053
26	exp online behavior/	1187
27	exp "social media use"/	1359

28	digital.ti,ab.	45629
29	exp computer games/	11603
30	gaming.ti,ab.	7769
31	"video games".ti,ab.	4793
32	"computer games".ti,ab.	1312
33	exp online community/	2949
34	"online communities".ti,ab.	1568
35	22 or 23 or 24 or 25 or 26 or 27 or 28 or 29 or 30 or 31 or 32 or 33 or 34	226213
36	exp Mental Health/	112171
37	"mental health".ti,ab.	265470
38	exp Well Being/	75583
39	wellbeing.ti,ab.	27510
40	"well-being".ti,ab.	126165
41	exp Anxiety Disorders/ or exp Anxiety/	142308
42	exp "Depression (Emotion)"/	27594
43	anxiety.ti,ab.	256921
44	anxious.ti,ab.	26038
45	depressi*.ti,ab.	372276
46	exp Psychological Stress/	10253
47	"psychological distress".ti,ab.	27444
48	exp Nonsuicidal Self-Injury/	8982
49	"self-harm".ti,ab.	7860
50	"self harm".ti,ab.	7860
51	exp Suicidal Ideation/	15011
52	"suicidal ideation".ti,ab.	15899
53	"mood disorder*".ti,ab.	18914
54	exp stress/	166502
55	"stress*".ti,ab.	336715
56	"psychological stress".ti,ab.	5897
57	36 or 37 or 38 or 39 or 40 or 41 or 42 or 43 or 44 or 45 or 46 or 47 or 48 or 49 or 50 or 51 or 52 or 53 or 54 or 55 or 56	1113747
58	exp longitudinal studies/	17570
59	Longitudinal.ti,ab.	158800
60	Longitudinal study.ti,ab.	51914

61	exp cohort analysis/	1801
62	Cohort study.ti,ab.	31132
63	"panel study".ti,ab.	3378
64	exp prospective studies/	1374
65	Prospective study.ti,ab.	13345
66	follow-up.ti,ab.	153931
67	58 or 59 or 60 or 61 or 62 or 63 or 64 or 65 or 66	330731
68	21 and 35 and 57 and 67	39

Results (39)

1. Demirgul, Suleyman Agah, Demetrovics, Zsolt, Czako, Andrea, Paksi, Borbala, Kokonyei, Gyongyi, Bothe, Beata. Journal of Sex Research. 2025 [cited Aug 18, 2025];No Pagination Specified. In: APA PsycInfo [Internet].
http://ovidsp.ovid.com/ovidweb.cgi?T=JS&PAGE=reference&D=psyc24&NEWS=N&AN=2026-54892-001

2. Wilson, Michael J, Fisher, Krista, Seidler, Zac. Masculinities and mental health in young men: From echo chambers to evidence. New York, NY. Palgrave Macmillan/Springer Nature. 2024.

3. Lawrence, Timothy I, Wojciechowski, Thomas, Lee, Kelly, Amador, Briana. Child Psychiatry and Human Development. 2025 [cited Mar 5, 2025];No Pagination Specified. In: APA PsycInfo [Internet].
http://ovidsp.ovid.com/ovidweb.cgi?T=JS&PAGE=reference&D=psyc24&NEWS=N&AN=2025-91646-001

4. Hayes, Samantha, Lonergan, Alexandra, Trompeter, Nora, Bussey, Kay, Hay, Phillipa, Mitchison, Deborah. Eating and Weight Disorders. 2022 [cited Dec 2022];27(8):3095-3108. In: APA PsycInfo [Internet].
http://ovidsp.ovid.com/ovidweb.cgi?T=JS&PAGE=reference&D=psyc24&NEWS=N&AN=2023-35498-009

5. Dollimore, Kora, Hurst, Megan, Cassarly, Jennifer A, Bell, Beth T. Psychology of Popular Media. 2025 [cited Jul 2025];14(3):478-483. In: APA PsycInfo [Internet].
http://ovidsp.ovid.com/ovidweb.cgi?T=JS&PAGE=reference&D=psyc23&NEWS=N&AN=2025-09086-001

6. Noll, Jennie G, Roitman, Margalit. Innovative methods in child maltreatment research and practice: Advances in detection, causal estimation, and intervention. Cham, Switzerland. Springer Nature Switzerland AG, Switzerland. 2023.

7. Miranda-Mendizabal, Andrea, Castellvi, Pere, Vilagut, Gemma, Alayo, Itxaso, Almenara, Jose, Ballester, Laura, Echeburua, Enrique, Gabilondo, Andrea, Gili, Margalida, Mortier, Philippe, Piqueras, Jose Antonio, Roca, Miquel, Auerbach, Randy P, Bruffaerts, Ronny, Kessler, Ronald C, Alonso Caballero, Jordi. Journal of Affective Disorders. 2024 [cited May 15, 2024];353. 52-59. In: APA PsycInfo [Internet].
http://ovidsp.ovid.com/ovidweb.cgi?T=JS&PAGE=reference&D=psyc23&NEWS=N&AN=2024-68154-009

8. Park, Jennifer J, King, Daniel L, Wilkinson-Meyers, Laura, Rodda, Simone N. JMIR Mental Health. 2022 [cited Sep 2022];9(9):1-11. In: APA PsycInfo [Internet]. http://ovidsp.ovid.com/ovidweb.cgi?T=JS&PAGE=reference&D=psyc23&NEWS=N&AN=2023-83566-005
9. Francioli, Stephane P, Shakeri, Angela, North, Michael S. PNAS Proceedings of the National Academy of Sciences of the United States of America. 2024 [cited Jun 17, 2024];121(26):In: APA PsycInfo [Internet]. http://ovidsp.ovid.com/ovidweb.cgi?T=JS&PAGE=reference&D=psyc23&NEWS=N&AN=2024-99979-001
10. Otmar, Christopher D, Merolla, Andy J. Health Communication. 2024 [cited Sep 1, 2024];No Pagination Specified. In: APA PsycInfo [Internet]. http://ovidsp.ovid.com/ovidweb.cgi?T=JS&PAGE=reference&D=psyc23&NEWS=N&AN=2025-21721-001
11. Solvberg, Nina, Torstveit, Monica Klungland, Solstad, Bard Erlend, Mountjoy, Margo, Rosenvinge, Jan H, Pettersen, Gunn, Sundgot-Borgen, Jorunn. Child Abuse & Neglect. 2024 [cited Jan 2024];147. 1-11. In: APA PsycInfo [Internet]. http://ovidsp.ovid.com/ovidweb.cgi?T=JS&PAGE=reference&D=psyc23&NEWS=N&AN=2024-68345-001
12. Mud Shukri, Muhammad Ikhwan, Baharom, Anisah. PLoS ONE. 2023 [cited Dec 20, 2023];18(12):In: APA PsycInfo [Internet]. http://ovidsp.ovid.com/ovidweb.cgi?T=JS&PAGE=reference&D=psyc23&NEWS=N&AN=2024-41878-001
13. No authorship indicated. Psychology of Sexual Orientation and Gender Diversity. 2024 [cited Jun 2024];11(2):304. In: APA PsycInfo [Internet]. http://ovidsp.ovid.com/ovidweb.cgi?T=JS&PAGE=reference&D=psyc22&NEWS=N&AN=2023-97850-001
14. Egorov, A. Yu. Neuroscience and Behavioral Physiology. 2023 [cited Sep 2023];53(7):1101-1106. In: APA PsycInfo [Internet]. http://ovidsp.ovid.com/ovidweb.cgi?T=JS&PAGE=reference&D=psyc22&NEWS=N&AN=2024-25739-001
15. Haas, Stephen M, Lannutti, Pamela J. Sexuality Research & Social Policy: A Journal of the NSRC. 2024 [cited Mar 2024];21(1):62-75. In: APA PsycInfo [Internet]. http://ovidsp.ovid.com/ovidweb.cgi?T=JS&PAGE=reference&D=psyc22&NEWS=N&AN=2023-92295-001
16. Baby, Anish. Internet pornography use, loneliness, and adult attachment patterns among Asian Indian young adults: An explanatory sequential mixed methods design. [Dissertation]. [Baby, Anish: St. Mary's University (Texas), Counselor Education and Supervision, US]; 2023. No Pagination Specified p.
17. Lee, Yu-Hao, Chen, Mo. Psychology of Popular Media. 2023 [cited Oct 2023];12(4):383-392. In: APA PsycInfo [Internet]. http://ovidsp.ovid.com/ovidweb.cgi?T=JS&PAGE=reference&D=psyc21&NEWS=N&AN=2022-95082-001
18. Wright, Michelle F, Wachs, Sebastian. Journal of Children and Media. 2022 [cited Aug 2022];16(3):451-460. In: APA PsycInfo [Internet]. http://ovidsp.ovid.com/ovidweb.cgi?T=JS&PAGE=reference&D=psyc21&NEWS=N&AN=2022-33951-001

19. Igarashi, Yurie, Staples, Jennifer, Vigil, Sabrina, Pero, Gabrielle, Gardner, Richard, Thomat, Ana, Abascal, Liana. Journal of Contextual Behavioral Science. 2022 [cited Apr 2022];24. 42-50. In: APA PsycInfo [Internet]. http://ovidsp.ovid.com/ovidweb.cgi?T=JS&PAGE=reference&D=psyc21&NEWS=N&AN=2022-47057-001
20. Shepherd, Benjamin F, Chang, Cindy J, Dyar, Christina, Brochu, Paula M, Selby, Edward A, Feinstein, Brian A. Psychology of Sexual Orientation and Gender Diversity. 2024 [cited Jun 2024];11(2):294-304. In: APA PsycInfo [Internet]. http://ovidsp.ovid.com/ovidweb.cgi?T=JS&PAGE=reference&D=psyc21&NEWS=N&AN=2022-88369-001
21. Kovacic Petrovic, Zrnka, Peraica, Tina, Kozaric-Kovacic, Dragica, Palavra, Irena Rojnic. Current Opinion in Psychiatry. 2022 [cited Sep 2022];35(5):324-331. In: APA PsycInfo [Internet]. http://ovidsp.ovid.com/ovidweb.cgi?T=JS&PAGE=reference&D=psyc21&NEWS=N&AN=2023-55953-006
22. Daskaluk, Samantha. #metoo: Effects of cyber sexual aggression victimization on women's health and relationship quality. [Dissertation]. [Daskaluk, Samantha: University of Windsor (Canada), PSYCHOLOGY, Canada]; 2022. No Pagination Specified p.
23. Zhang, Jiwen, Lo, Herman Hayming, Au, Alma Maylan. Journal of Affective Disorders. 2021 [cited Mar 15, 2021];283. 335-343. In: APA PsycInfo [Internet]. http://ovidsp.ovid.com/ovidweb.cgi?T=JS&PAGE=reference&D=psyc20&NEWS=N&AN=2021-26411-046
24. Kittleman, Melinda M. More than just words on a screen: A biopsychosocial approach to understanding effects of race-related media. [Dissertation]. [Kittleman, Melinda M.: University of Missouri - Saint Louis, Psychology, US]; 2021. No Pagination Specified p.
25. Copp, Jennifer E, Mumford, Elizabeth A, Taylor, Bruce G. Journal of Adolescence. 2021 [cited Dec 2021];93. 202-211. In: APA PsycInfo [Internet]. http://ovidsp.ovid.com/ovidweb.cgi?T=JS&PAGE=reference&D=psyc20&NEWS=N&AN=2022-24056-017
26. Kuss, Daria J, Kristensen, Anne Marie, Lopez-Fernandez, Olatz. Computers in Human Behavior. 2021 [cited Feb 2021];115. In: APA PsycInfo [Internet]. http://ovidsp.ovid.com/ovidweb.cgi?T=JS&PAGE=reference&D=psyc20&NEWS=N&AN=2020-97437-001
27. Chao, Cheng-Min, Yu, Tai-Kuei. International Journal of Mental Health and Addiction. 2021 [cited Aug 25, 2021];No Pagination Specified. In: APA PsycInfo [Internet]. http://ovidsp.ovid.com/ovidweb.cgi?T=JS&PAGE=reference&D=psyc20&NEWS=N&AN=2021-80384-001
28. Maroney, Meredith R. Exploring the efficacy of an online intervention in processing experiences of heterosexism among autistic-LGBTQ individuals. [Dissertation]. [Maroney, Meredith R.: University of Massachusetts Boston, Counseling and School Psychology (PhD), US]; 2021. No Pagination Specified p.
29. Hirschtritt, Matthew E, Tucker, Douglas, Binder, Renee L. Journal of the American Academy of Psychiatry and the Law. 2019 [cited Jun 1, 2019];47(2):155-164. In: APA PsycInfo [Internet]. http://ovidsp.ovid.com/ovidweb.cgi?T=JS&PAGE=reference&D=psyc18&NEWS=N&AN=2020-03660-001

30. Kuhn, Simone, Kugler, Dimitrij Tycho, Schmalen, Katharina, Weichenberger, Markus, Witt, Charlotte, Gallinat, Jurgen. Molecular Psychiatry. 2019 [cited Aug 2019];24(8):1220-1234. In: APA PsycInfo [Internet].
http://ovidsp.ovid.com/ovidweb.cgi?T=JS&PAGE=reference&D=psyc18&NEWS=N&AN=2019-39734-001
31. Ma, Cecilia M. S. Applied Research in Quality of Life. 2019 [cited Apr 15, 2019];14(2):423-439. In: APA PsycInfo [Internet].
http://ovidsp.ovid.com/ovidweb.cgi?T=JS&PAGE=reference&D=psyc18&NEWS=N&AN=2018-13297-001
32. Werner, Marlene, Stulhofer, Aleksandar, Waldorp, Lourens, Jurin, Tanja. Journal of Sexual Medicine. 2018 [cited Apr 2018];15(4):612. In: APA PsycInfo [Internet].
http://ovidsp.ovid.com/ovidweb.cgi?T=JS&PAGE=reference&D=psyc17&NEWS=N&AN=2018-14553-025
33. Werner, Marlene, Stulhofer, Aleksandar, Waldorp, Lourens, Jurin, Tanja. Journal of Sexual Medicine. 2018 [cited Mar 2018];15(3):373-386. In: APA PsycInfo [Internet].
http://ovidsp.ovid.com/ovidweb.cgi?T=JS&PAGE=reference&D=psyc17&NEWS=N&AN=2018-10167-016
34. Ke, Guek Nee, Wong, Siew Fan. Journal of Rational-Emotive & Cognitive-Behavior Therapy. 2018 [cited Jun 2018];36(2):187-200. In: APA PsycInfo [Internet].
http://ovidsp.ovid.com/ovidweb.cgi?T=JS&PAGE=reference&D=psyc17&NEWS=N&AN=2017-51192-001
35. Grubbs, Joshua B, Wilt, Joshua A, Exline, Julie J, Pargament, Kenneth I, Kraus, Shane W. Addiction. 2018 [cited Mar 2018];113(3):496-506. In: APA PsycInfo [Internet].
http://ovidsp.ovid.com/ovidweb.cgi?T=JS&PAGE=reference&D=psyc17&NEWS=N&AN=2017-41260-001
36. McGinley, Meredith, Rospenda, Kathleen M, Liu, Li, Richman, Judith A. Journal of Adolescence. 2016 [cited Dec 2016];53. 152-163. In: APA PsycInfo [Internet].
http://ovidsp.ovid.com/ovidweb.cgi?T=JS&PAGE=reference&D=psyc15&NEWS=N&AN=2016-58515-016
37. Chang, Fong-Ching, Chiu, Chiung-Hui, Miao, Nae-Fang, Chen, Ping-Hung, Lee, Ching-Mei, Chiang, Jeng-Tung. Journal of Health Psychology. 2016 [cited Jun 2016];21(6):1107-1118. In: APA PsycInfo [Internet].
http://ovidsp.ovid.com/ovidweb.cgi?T=JS&PAGE=reference&D=psyc15&NEWS=N&AN=2016-29381-023
38. Huffman, Ann Hergatt, Olson, Kristine J, O'Gara, Thomas C Jr., King, Eden B. Journal of Managerial Psychology. 2014 [cited 2014];29(7):774-793. In: APA PsycInfo [Internet].
http://ovidsp.ovid.com/ovidweb.cgi?T=JS&PAGE=reference&D=psyc13&NEWS=N&AN=2014-40394-001
39. Ybarra, Michele L, Mitchell, Kimberly J. CyberPsychology & Behavior. 2005 [cited Oct 2005];8(5):473-486. In: APA PsycInfo [Internet].
http://ovidsp.ovid.com/ovidweb.cgi?T=JS&PAGE=reference&D=psyc5&NEWS=N&AN=2005-13350-008

Web of Science

Search terms

TS=(misogyn* OR "sexual harassment" OR sexi* OR chauvin* OR bigot* OR prejudice OR "revenge porn" OR incel OR "gendered hate" OR trolling OR "intimate image" OR porn OR manosphere) AND TS=(online OR "social media" OR internet OR digital OR gaming OR "video games" OR "online communities") AND TS=("mental health" OR wellbeing OR "well-being" OR anxiety OR depression OR depressi* OR "psychological distress" OR "self-harm" OR "suicidal ideation" OR "mood disorder*" OR stress) AND TS=(longitudinal OR "longitudinal study" OR "cohort study" OR "panel study" OR "prospective study" OR "follow-up study")

Results (29)

Record 1 of 29

Title: Exposure of medical students to sexism and sexual harassment and their association with mental health: a cross-sectional study at a Swiss medical school

Author(s): Barbier, JM (Barbier, Jeanne Marie); Carrard, V (Carrard, Valerie); Schwarz, J (Schwarz, Joelle); Berney, S (Berney, Sylvie); Clair, C (Clair, Carole); Berney, A (Berney, Alexandre)

Source: BMJ OPEN **Volume:** 13 **Issue:** 4 **Article Number:** e069001 **DOI:** 10.1136/bmjopen-2022-069001 **Published Date:** 2023 APR

Accession Number: WOS:001057956000089

PubMed ID: 37105707

Author Identifiers:

Author	Web of Science ResearcherID	ORCID Number
Schwarz, Joelle		0000-0002-0992-3165
Carrard, Valerie		0000-0001-7355-9567
Berney, Sylvie		0000-0001-7099-6999
Clair, Carole	D-9786-2014	0000-0001-5281-0943
Berney, Alexandre		0000-0001-9535-1222
Clair, Carole	AAF-6675-2021	
Barbier, Jeanne		0000-0001-8259-1761

ISSN: 2044-6055

Record 2 of 29

Title: Cross-Sectional and Longitudinal Relationships Between Young Student Women's Experiences of Everyday Sexual Harassment on Social Media and Self-Objectification, Body Shame, and Personal Safety Anxiety

Author(s): Dollimore, K (Dollimore, Kora); Hurst, M (Hurst, Megan); Cassarly, JA (Cassarly, Jennifer A.); Bell, BT (Bell, Beth T.)

Source: PSYCHOLOGY OF POPULAR MEDIA **Volume:** 14 **Issue:** 3 **Pages:** 478-483 **DOI:** 10.1037/ppm0000554 **Early Access Date:** AUG 2024 **Published Date:** 2025 JUL

Accession Number: WOS:001300805900001

Author Identifiers:

Author	Web of Science ResearcherID	ORCID Number
Bell, Beth		0000-0002-6587-0336
Cassarly, Jennifer		0000-0003-1177-2519
Hurst, Megan		0000-0002-2662-3615

ISSN: 2689-6567

eISSN: 2689-6575

Record 3 of 29

Title: It isn't all just fun and games: Collegiate participation in extracurricular activities and risk for generalized and sexual harassment, psychological distress, and alcohol use

Author(s): McGinley, M (McGinley, Meredith); Rospenda, KM (Rospenda, Kathleen M.); Liu, L (Liu, Li); Richman, JA (Richman, Judith A.)

Source: JOURNAL OF ADOLESCENCE **Volume:** 53 **Pages:** 152-163 **DOI:** 10.1016/j.adolescence.2016.10.001 **Published Date:** 2016 DEC

Accession Number: WOS:000389092600016

PubMed ID: 27771523

Author Identifiers:

Author	Web of Science ResearcherID	ORCID Number
Rospenda, Kathy	ABF-1383-2020	
McGinley, Meredith		0000-0002-9542-9162

ISSN: 0140-1971

eISSN: 1095-9254

Record 4 of 29

Title: Prevalence and Correlates of Problematic Internet Experiences and Computer-Using Time: A Two-Year Longitudinal Study in Korean School Children

Author(s): Yang, SJ (Yang, Su-Jin); Stewart, R (Stewart, Robert); Lee, JY (Lee, Ju-Yeon); Kim, JM (Kim, Jae-Min); Kim, SW (Kim, Sung-Wan); Shin, IS (Shin, Il-Seon); Yoon, JS (Yoon, Jin-Sang)

Source: PSYCHIATRY INVESTIGATION **Volume:** 11 **Issue:** 1 **Pages:** 24-31 **DOI:** 10.4306/pi.2014.11.1.24 **Published Date:** 2014 JAN

Accession Number: WOS:000330682500004

PubMed ID: 24605120

Author Identifiers:

Author	Web of Science ResearcherID	ORCID Number
shin, i	JCE-1227-2023	
Lee, Jung-Seok	L-6826-2019	

KIM, Jae-Min	S-7637-2019	
Stewart, Robert	B-1667-2010	0000-0002-4435-6397

ISSN: 1738-3684

eISSN: 1976-3026

Record 5 of 29

Title: Longitudinal relationship between experience of sexual harassment and 2-year body image and weight outcomes in adolescence: mediation through self-objectification and psychological distress

Author(s): Hayes, S (Hayes, Samantha); Lonergan, A (Lonergan, Alexandra); Trompeter, N (Trompeter, Nora); Bussey, K (Bussey, Kay); Hay, P (Hay, Phillipa); Mitchison, D (Mitchison, Deborah)

Source: EATING AND WEIGHT DISORDERS-STUDIES ON ANOREXIA BULIMIA AND OBESITY **Volume:** 27 **Issue:** 8 **Pages:** 3095-3108 **DOI:** 10.1007/s40519-022-01432-4 **Early Access Date:** JUL 2022 **Published Date:** 2022 DEC

Accession Number: WOS:000825175100001

PubMed ID: 35834106

Author Identifiers:

Author	Web of Science ResearcherID	ORCID Number
Trompeter, Nora		0000-0001-5800-8679
Hayes, Samantha	AAS-6064-2021	
Mitchison, Deborah	AAH-4842-2021	
MITCHISON, DEBORAH		0000-0002-6736-7937
Hayes, Samantha		0000-0002-1182-3913
Trompeter, Nora	W-5126-2019	
Bussey, Kay		0000-0001-6806-0892

ISSN: 1124-4909

eISSN: 1590-1262

Record 6 of 29

Title: Pornography Use and Depressive Symptoms: Examining the Role of Moral Incongruence

Author(s): Perry, SL (Perry, Samuel L.)

Source: SOCIETY AND MENTAL HEALTH **Volume:** 8 **Issue:** 3 **Pages:** 195-213 **DOI:** 10.1177/2156869317728373 **Published Date:** 2018 NOV

Accession Number: WOS:000450309600002

Author Identifiers:

Author	Web of Science ResearcherID	ORCID Number
Perry, Samuel		0000-0002-6398-636X

ISSN: 2156-8693

eISSN: 2156-8731

Record 7 of 29

Title: Longitudinal Comorbidity of Anxiety and Depressive Symptoms: The Risk of Cyber Sexual Harassment

Author(s): Lawrence, TI (Lawrence, Timothy I.); Wojciechowski, T (Wojciechowski, Thomas); Lee, K (Lee, Kelly); Amador, B (Amador, Briana)

Source: CHILD PSYCHIATRY & HUMAN DEVELOPMENT **DOI:** 10.1007/s10578-025-01820-8 **Early Access Date:** MAR 2025 **Published Date:** 2025 MAR 5

Accession Number: WOS:001437491300001

PubMed ID: 40042776

ISSN: 0009-398X

eISSN: 1573-3327

Record 8 of 29

Title: Does playing violent video games cause aggression? A longitudinal intervention study

Author(s): Kühn, S (Kuehn, Simone); Kugler, DT (Kugler, Dimitrij Tycho); Schmalen, K (Schmalen, Katharina); Weichenberger, M (Weichenberger, Markus); Witt, C (Witt, Charlotte); Gallinat, J (Gallinat, Juergen)

Source: MOLECULAR PSYCHIATRY **Volume:** 24 **Issue:** 8 **Pages:** 1220-1234 **DOI:** 10.1038/s41380-018-0031-7 **Published Date:** 2019 AUG

Accession Number: WOS:000476715100009

Author Identifiers:

Author	Web of Science ResearcherID	ORCID Number
Kuhn, Simone		0000-0001-6823-7969
Kuehn, Simone	P-6145-2014	

ISSN: 1359-4184

eISSN: 1476-5578

Record 9 of 29

Title: Problematic online gaming, subjective health complaints, and depression among adolescent gamers from the United States: the role of console-gaming aggression

Author(s): Wright, MF (Wright, Michelle F.); Wachs, S (Wachs, Sebastian)

Source: JOURNAL OF CHILDREN AND MEDIA **Volume:** 16 **Issue:** 3 **Pages:** 451-460 **DOI:** 10.1080/17482798.2022.2036211 **Early Access Date:** FEB 2022 **Published Date:** 2022 JUL 3

Accession Number: WOS:000755109600001

Author Identifiers:

Author	Web of Science ResearcherID	ORCID Number
Wachs, Sebastian		0000-0003-2787-6646
Wright, Michelle		0000-0002-6703-8586

ISSN: 1748-2798

eISSN: 1748-2801

Record 10 of 29

Title: Online sexual harassment and cyberbullying in a nationally representative sample of teens: Prevalence, predictors, and consequences

Author(s): Copp, JE (Copp, Jennifer E.); Mumford, EA (Mumford, Elizabeth A.); Taylor, BG (Taylor, Bruce G.)

Source: JOURNAL OF ADOLESCENCE **Volume:** 93 **Issue:** 1 **Pages:** 202-211 **DOI:** 10.1016/j.adolescence.2021.10.003 **Published Date:** 2021 DEC

Accession Number: WOS:000768572700002

PubMed ID: 34801812

ISSN: 0140-1971

eISSN: 1095-9254

Record 11 of 29

Title: Intergroup Contact With a Virtual Refugee: Reducing Prejudice Through a Cooperative Game

Author(s): Lee, YH (Lee, Yu-Hao); Chen, M (Chen, Mo)

Source: PSYCHOLOGY OF POPULAR MEDIA **Volume:** 12 **Issue:** 4 **Pages:** 383-392 **DOI:** 10.1037/ppm0000433 **Early Access Date:** SEP 2022 **Published Date:** 2023 OCT

Accession Number: WOS:000847817400001

Author Identifiers:

Author	Web of Science ResearcherID	ORCID Number
Chen, Mo		0000-0002-2036-4130
Lee, Yu-Hao		0000-0002-0915-7616
Lee, Yu-Hao	MGU-4870-2025	

ISSN: 2689-6567

eISSN: 2689-6575

Record 12 of 29

Title: Nonconsensual Dissemination of Sexual Images Among Adolescents: Associations With Depression and Self-Esteem

Author(s): Sciacca, B (Sciacca, Beatrice); Mazzone, A (Mazzone, Angela); Loftsson, M (Loftsson, Magnus); Norman, JO (O'Higgins Norman, James); Foody, M (Foody, Mairead)

Source: JOURNAL OF INTERPERSONAL VIOLENCE **Volume:** 38 **Issue:** 15-16 **Pages:** 9438-9464 **DOI:** 10.1177/08862605231165777 **Early Access Date:** APR 2023 **Published Date:** 2023 AUG

Accession Number: WOS:000972066400001

PubMed ID: 37073923

Author Identifiers:

Author	Web of Science ResearcherID	ORCID Number

Mazzone, Angela	AAB-8667-2020	
Foody, Mairead		0000-0003-0026-0294
Sciacca, Beatrice	LZI-0303-2025	
Foody, Mairead	KLC-3270-2024	
Sciacca, Beatrice		0000-0003-0678-4902
Mazzone, Angela		0000-0002-5858-8033

ISSN: 0886-2605

eISSN: 1552-6518

Record 13 of 29

Title: The Incidence and Risk Factors of Workplace Violence towards Female Nurses Reported via Internet in an Acute Psychiatric Hospital

Author(s): Chen, WC (Chen, Wen-Ching); Huang, CJ (Huang, Chuan-Ju); Chen, CC (Chen, Chiao-Chicy); Wang, JD (Wang, Jung-Der)

Source: ARCHIVES OF ENVIRONMENTAL & OCCUPATIONAL HEALTH **Volume:** 66 **Issue:** 2 **Pages:** 100-106 **DOI:** 10.1080/19338244.2010.511310 **Published Date:** 2011

Accession Number: WOS:000299993700006

PubMed ID: 24484367

Author Identifiers:

Author	Web of Science ResearcherID	ORCID Number
Chen, Pao-Huan	ABG-5963-2020	

ISSN: 1933-8244

eISSN: 2154-4700

Record 14 of 29

Title: The Dynamic Nature of Coping with Gender Discrimination: Appraisals, Strategies and Well-being Over Time

Author(s): Foster, M (Foster, Mindi)

Source: SEX ROLES **Volume:** 60 **Issue:** 9-10 **Pages:** 694-707 **DOI:** 10.1007/s11199-008-9568-2 **Published Date:** 2009 MAY

Accession Number: WOS:000265308200007

Author Identifiers:

Author	Web of Science ResearcherID	ORCID Number
Foster, Mindi	KPY-4747-2024	

ISSN: 0360-0025

Record 15 of 29

Title: Cross-sectional study of the rates of military sexual trauma (MST) and associations with adverse mental health outcomes among UK female ex-service personnel: a study protocol

Author(s): Obradovic, T (Obradovic, Tamara); Murphy, D (Murphy, Dominic); Fear, NT (Fear, Nicola T.); Sharp, ML (Sharp, Marie-Louise)

Source: BMJ OPEN **Volume:** 15 **Issue:** 6 **Article Number:** e096912 **DOI:** 10.1136/bmjopen-2024-096912 **Published Date:** 2025 JUN 26

Accession Number: WOS:001519645600001

PubMed ID: 40578887

Author Identifiers:

Author	Web of Science ResearcherID	ORCID Number
Fear, Nicola		0000-0002-5792-2925
Sharp, Marie-Louise	HNI-7561-2023	
Fear, Nicola	A-1917-2012	

ISSN: 2044-6055

Record 16 of 29

Title: How Do You Feel Online? Exploiting Smartphone Sensors to Detect Transitory Emotions during Social Media Use

Author(s): Ruensuk, M (Ruensuk, Mintra); Cheon, E (Cheon, Eunyong); Hong, H (Hong, Hwajung); Oakley, I (Oakley, Ian)

Source: PROCEEDINGS OF THE ACM ON INTERACTIVE MOBILE WEARABLE AND UBIQUITOUS TECHNOLOGIES-IMWUT **Volume:** 4 **Issue:** 4 **DOI:** 10.1145/3432223 **Published Date:** 2020 DEC

Accession Number: WOS:000908400400035

Author Identifiers:

Author	Web of Science ResearcherID	ORCID Number
Oakley, Ian	B-2103-2013	

eISSN: 2474-9567

Record 17 of 29

Title: Risk factors for sexual harassment and abuse victimization among adolescent athletes and non-athletes: A one-year follow-up study

Author(s): Solvberg, N (Solvberg, Nina); Torstveit, MK (Torstveit, Monica Klungland); Solstad, BE (Solstad, Bard Erlend); Mountjoy, M (Mountjoy, Margo); Rosenvinge, JH (Rosenvinge, Jan H.); Pettersen, G (Pettersen, Gunn); Sundgot-Borgen, J (Sundgot-Borgen, Jorunn)

Source: CHILD ABUSE & NEGLECT **Volume:** 147 **Article Number:** 106592 **DOI:** 10.1016/j.chiabu.2023.106592 **Early Access Date:** DEC 2023 **Published Date:** 2024 JAN

Accession Number: WOS:001140808900001

PubMed ID: 38113571

Author Identifiers:

Author	Web of Science ResearcherID	ORCID Number

Sølvberg, Nina	KII-4923-2024	

ISSN: 0145-2134

eISSN: 1873-7757

Record 18 of 29

Title: Fostering Empathy Through Play: The Impact of <i>Far From Home</i> on University Staff's Understanding of International Students

Author(s): Shan, SS (Shan, Shuanghui Sofia); Illingworth, S (Illingworth, Sam)

Source: BEHAVIORAL SCIENCES **Volume:** 15 **Issue:** 6 **Article Number:** 820 **DOI:** 10.3390/bs15060820 **Published Date:** 2025 JUN 14

Accession Number: WOS:001516175200001

PubMed ID: 40564602

Author Identifiers:

Author	Web of Science ResearcherID	ORCID Number
Illingworth, Sam	A-4899-2015	

eISSN: 2076-328X

Record 19 of 29

Title: A Comparative Study of the Help-Seeking Behavior of Victims of Contact Sexual Violence and Image-Based Sexual Harassment and Abuse

Author(s): Pijlman, V (Pijlman, Valerie); Boertien, E (Boertien, Erin)

Source: VIOLENCE AGAINST WOMEN **DOI:** 10.1177/10778012241283496 **Early Access Date:** SEP 2024 **Published Date:** 2024 SEP 27

Accession Number: WOS:001320865300001

PubMed ID: 39327999

Author Identifiers:

Author	Web of Science ResearcherID	ORCID Number
Pijlman, Valerie		0000-0001-7422-0389

ISSN: 1077-8012

eISSN: 1552-8448

Record 20 of 29

Title: Testing the Effectiveness of an Intervention to Improve Romanian Teachers' LGBT+-Related Attitudes, Cognitions, Behaviors, and Affect: Protocol for a Randomized Controlled Trial

Author(s): Latu, I (Latu, Ioana); Salagean, N (Salagean, Nastasia); Larsen, TMB (Larsen, Torill M. B.); Isbasoiu, AB (Isbasoiu, Andreea Bogdana); Sava, FA (Sava, Florin Alin)

Source: JMIR RESEARCH PROTOCOLS **Volume:** 13 **Article Number:** e54254 **DOI:** 10.2196/54254 **Published Date:** 2024

Accession Number: WOS:001223005900004

PubMed ID: 38652533

Author Identifiers:

Author	Web of Science ResearcherID	ORCID Number
Salagean, Nastasia	KFQ-4020-2024	
Larsen, Torill M.B		0000-0003-3457-9922
Isbasoiu, Andreea		0000-0003-0377-9358
Larsen, Torill	AAG-9738-2020	
Latu, Ioana		0000-0003-3328-9261
Sava, Florin	C-1427-2011	0000-0001-8898-1306
Salagean, Nastasia		0000-0002-9923-1559

ISSN: 1929-0748

Record 21 of 29

Title: Associations of Sexual Orientation-Related Minority and Military Stressors With Past-Year Cigarette Use Among Lesbian, Gay, and Bisexual Veterans

Author(s): Ruiz, RA (Ruiz, Raymond A.); Lehavot, K (Lehavot, Keren); Heffner, JL (Heffner, Jaimee L.); Kava, CM (Kava, Christine M.); Ornelas, IJ (Ornelas, India J.)

Source: NICOTINE & TOBACCO RESEARCH **Volume:** 26 **Issue:** 7 **Pages:** 852-860 **DOI:** 10.1093/ntr/ntad210 **Early Access Date:** NOV 2023 **Published Date:** 2023 NOV 10

Accession Number: WOS:001100447700001

PubMed ID: 37878537

Author Identifiers:

Author	Web of Science ResearcherID	ORCID Number
Ruiz, Raymond	LDG-3100-2024	
Kava, Christine Marie		0000-0003-4634-3837
Kava, Marie	HHN-7375-2022	
Ruiz, Raymond		0000-0002-6827-9285
Heffner, Jaimee		0000-0001-9604-5489

ISSN: 1462-2203

eISSN: 1469-994X

Record 22 of 29

Title: Peer victimization and Internet addiction among adolescents: The role of anger rumination and social dominance orientation

Author(s): Chen, JH (Chen, Jiahui); Xiong, YK (Xiong, Yuke); Wang, QQ (Wang, Quanquan); Xu, XF (Xu, Xiaofeng); Qin, XN (Qin, Xingna); Ren, P (Ren, Ping)

Source: JOURNAL OF ADOLESCENCE **Volume:** 95 **Issue:** 8 **Pages:** 1578-1589 **DOI:** 10.1002/jad.12227 **Early Access Date:** AUG 2023 **Published Date:** 2023 DEC

Accession Number: WOS:001040077000001

PubMed ID: 37538030

Author Identifiers:

Author	Web of Science ResearcherID	ORCID Number
Qin, Xingna		0000-0002-3662-8348

ISSN: 0140-1971

eISSN: 1095-9254

Record 23 of 29

Title: "They are Giving Folks Permission to Discriminate and Hate": A 4-Year Longitudinal Analysis of Perceived Impact of the Trump Administration on LGBTQ plus Individuals and Relationships, 2017-2020

Author(s): Haas, SM (Haas, Stephen M.); Lannutti, PJ (Lannutti, Pamela J.)

Source: SEXUALITY RESEARCH AND SOCIAL POLICY **Volume:** 21 **Issue:** 1 **Pages:** 62-75 **DOI:** 10.1007/s13178-023-00843-x **Early Access Date:** JUL 2023 **Published Date:** 2024 MAR

Accession Number: WOS:001030503800001

Author Identifiers:

Author	Web of Science ResearcherID	ORCID Number
Lannutti, Pamela		0000-0003-1547-9205

ISSN: 1868-9884

eISSN: 1553-6610

Record 24 of 29

Title: Income-related health inequalities: does perceived discrimination matter?

Author(s): Simons, AMW (Simons, Audrey Maria Wilhelmina); Groffen, DAI (Groffen, Danielle Adriana Irene); Bosma, H (Bosma, Hans)

Source: INTERNATIONAL JOURNAL OF PUBLIC HEALTH **Volume:** 58 **Issue:** 4 **Pages:** 513-520 **DOI:** 10.1007/s00038-012-0429-y **Published Date:** 2013 AUG

Accession Number: WOS:000322117400005

Author Identifiers:

Author	Web of Science ResearcherID	ORCID Number
Simons, Audrey		0000-0002-8030-5247
Bosma, Hans	A-6184-2013	
Groffen, Danielle		0000-0002-9685-3109

ISSN: 1661-8556

eISSN: 1661-8564

Record 25 of 29

Title: One in Ten Adolescents and Young Adults Report Changes in Their Sexual Orientation Identity Over 2-Month Follow-Up: Results From a Longitudinal Cohort Study

Author(s): Ranker, LR (Ranker, Lynsie R.); Korkodilos, R (Korkodilos, R.); Conti, J (Conti, Jennifer); Nelson, KM (Nelson, Kimberly M.); Xuan, ZM (Xuan, Ziming); Gordon, AR (Gordon, Allegra R.); Katz-Wise, SL (Katz-Wise, Sabra L.)

Source: PSYCHOLOGY OF SEXUAL ORIENTATION AND GENDER DIVERSITY **DOI:** 10.1037/sgd0000782 **Early Access Date:** JAN 2025 **Published Date:** 2025 JAN 2

Accession Number: WOS:001386418500001

Author Identifiers:

Author	Web of Science ResearcherID	ORCID Number
Ranker, Lynsie		0000-0001-7928-2781
Korkodilos, Rachel		0000-0003-0304-2188
Katz-Wise, Sabra	AGY-0152-2022	

ISSN: 2329-0382

eISSN: 2329-0390

Record 26 of 29

Title: Effect of a Narrative-Based Online Course Aimed at Reducing Stigma Toward Transgender Children and Adolescents: Longitudinal Observational Study

Author(s): Greuel, M (Greuel, Merlin); Nguyen, V (Nguyen, Van Kinh); Amsalem, D (Amsalem, Doron); Adam, M (Adam, Maya); Baernighausen, T (Baernighausen, Till)

Source: JMIR FORMATIVE RESEARCH **Volume:** 9 **Article Number:** e59605 **DOI:** 10.2196/59605 **Published Date:** 2025

Accession Number: WOS:001447195700003

PubMed ID: 39786893

Author Identifiers:

Author	Web of Science ResearcherID	ORCID Number
Adam, Maya		0000-0001-5563-421X
Amsalem, Doron	ABA-7896-2021	
Barnighausen, Till	Y-2388-2019	
Amsalem, Doron		0000-0002-8056-3211

eISSN: 2561-326X

Record 27 of 29

Title: Public Discourse Toward Older Drivers in Japan Using Social Media Data From 2010 to 2022: Longitudinal Analysis

Author(s): Nakanishi, A (Nakanishi, Akito); Ichikawa, M (Ichikawa, Masao); Sano, Y (Sano, Yukie)

Source: JMIR INFODEMIOLOGY **Volume:** 5 **Article Number:** e69321 **DOI:** 10.2196/69321 **Published Date:** 2025

Accession Number: WOS:001513476600001

PubMed ID: 40523233

eISSN: 2564-1891

Record 28 of 29

Title: Emotional Dysregulation and Coping Strategies in the Context of Problematic Pornography Use: A Narrative Review

Author(s): Testa, G (Testa, Giulia); Villena-Moya, A (Villena-Moya, Alejandro); Chiclana-Actis, C (Chiclana-Actis, Carlos)

Source: CURRENT ADDICTION REPORTS **Volume:** 11 **Issue:** 2 **Pages:** 229-241 **DOI:** 10.1007/s40429-024-00548-0 **Early Access Date:** MAR 2024 **Published Date:** 2024 APR

Accession Number: WOS:001175579300001

Author Identifiers:

Author	Web of Science ResearcherID	ORCID Number
Testa, Giulia		0000-0003-4200-8062
Testa, Giulia	AAE-6991-2022	
Villena-Moya, Alejandro		0000-0001-7240-4440
Chiclana-Actis, Carlos		0000-0001-7586-7045
Villena, Alejandro	GQP-1407-2022	

eISSN: 2196-2952

Record 29 of 29

Title: Research on multimorbidity in primary care. Selected abstracts from the EGPRN meeting in Tampere, Finland, 9-12 May 2019 All abstracts of the conference can be found at the EGPRN website: www.egprn.org/page/conference-abstracts

Author(s): [Anonymous] ([Anonymous])

Source: EUROPEAN JOURNAL OF GENERAL PRACTICE **Volume:** 25 **Issue:** 3 **Pages:** 164-175 **DOI:** 10.1080/13814788.2019.1643166 **Published Date:** 2019 JUL 3
